# Recent Advances in Black Phosphorus and Phosphorene Nanosheets: From Interface Engineering to Energy Storage, Catalysis, and Biomedicine

**DOI:** 10.3390/nano16201331

**Published:** 2026-10-21

**Authors:** Amin Rabiei Baboukani, Chunlei Wang

**Affiliations:** 1 Department of Mechanical and Materials Engineering, Florida International University, Miami, FL 33174, USA; 2 Department of Mechanical and Aerospace Engineering, University of Miami, Coral Gables, FL 33146, USA; wangc@miami.edu

**Keywords:** black phosphorus, phosphorene, two-dimensional materials, interface engineering, energy storage, electrocatalysis, photocatalysis, nanomedicine, biosafety

## Abstract

Black phosphorus (BP) and its few-layer counterpart, phosphorene, combine an anisotropic two-dimensional lattice, thickness-dependent electronic properties, strong near-infrared response, and chemically accessible phosphorus sites. The same surface chemistry that makes phosphorene attractive also makes it difficult to process and preserve reproducibly. This review focuses mainly on studies published from 2023 through September 2026 and examines how the field has shifted from proof-of-concept exfoliation toward deliberate interface control. Recent progress in liquid and electrochemical exfoliation, bipolar deposition, covalent functionalization, molecular passivation, and removable protective layers is discussed together with applications in Li- and Na-ion batteries, Li-S systems, supercapacitors, electrocatalytic water splitting, photocatalytic hydrogen evolution, CO_2_ reduction, drug delivery, phototherapy, tissue regeneration, antibacterial treatment, and biosafety. Across these areas, thickness, defect population, oxidation state, interfacial bonding, and nanosheet accessibility repeatedly determine both performance and failure. Particular attention is given to realistic electrochemical metrics, identification of catalytic active states, batch-to-batch reproducibility, and organ-specific safety. The review closes by outlining practical requirements for moving phosphorene from optimized laboratory demonstrations toward reproducible energy, catalytic, and biomedical technologies.

## 1. Introduction

Black phosphorus stands out among two-dimensional materials because its biggest strengths and its main weakness come from the same surface chemistry. The puckered phosphorus lattice gives rise to anisotropic charge transport, a thickness-dependent bandgap, a large accessible surface area, and highly reactive lone-pair electrons. Those features are exactly why BP and phosphorene have been explored for ion storage, catalysis, photothermal conversion, drug loading, and interfacial charge transfer. They are also the reason the material is difficult to preserve. Exposed phosphorene can oxidize quickly in the presence of oxygen and water, especially under light. In practice, then, the relevant material is not just “phosphorene” in the abstract, but phosphorene with a particular thickness, defect density, oxidation state, adsorbate history, interfacial environment, and storage record.

By 2021, most of the foundational exfoliation routes had already been demonstrated, and BP had been shown to work as an active material in batteries and supercapacitors. A previous review by Rabiei Baboukani and co-workers summarized liquid-phase exfoliation, anodic and cathodic electrochemical routes, bipolar electrochemistry, and the early electrochemical energy-storage literature [1]. That earlier review also highlighted several unresolved issues, including ambient degradation, incomplete control over size and thickness, large alloying-induced volume changes, limited full-cell validation, and an incomplete picture of how phosphorene interfaces evolve during operation [1]. The more useful question in 2026 is therefore not whether phosphorene can be prepared or whether it can store charge. The real question is what has changed enough to make the field more convincing today than it was during the first wave of proof-of-concept studies.

A number of recent reviews have tackled important parts of this landscape. Passivation strategies for few-layer BP battery anodes were summarized in 2023 [2]; broad synthesis, defect tailoring, and photocatalysis were reviewed in 2026 [3]; environmental, energy, and biomedical applications were surveyed in 2025 [4]; cancer-oriented BP nanoplatforms were analyzed in detail [5]; and biomedical uses were reviewed in 2024 [6]. More recently, an energy-storage-focused review consolidated BP across battery and supercapacitor chemistries [7], while a dedicated stability review organized physical and chemical passivation strategies [8]. Neighboring 2026 reviews on violet phosphorus and modern 2D heterostructures further illustrate how rapidly the broader phosphorus and interface-engineering literature is expanding [9,10]. These contributions are valuable, but they also make a conventional catalogue of “properties and applications” increasingly redundant. The present review is instead organized around a cross-application question: how does the chemical and structural state of the phosphorene interface evolve from synthesis to operation, and which measurements are needed to show that an apparent performance gain is physically meaningful and practically relevant?

This review therefore focuses on primary research published from January 2023 through September 2026, while using earlier studies only when they establish an essential benchmark or mechanistic framework. The distinctive feature is not the date range itself, but the comparison framework. Energy-storage studies are evaluated with electrode-level metrics such as active-material loading, initial Coulombic efficiency, areal capacity, electrolyte inventory, and full-cell evidence where these values are reported. Catalytic studies are discussed in terms of the chemical state that actually exists under bias or illumination, rather than assuming that the as-synthesized BP interface remains unchanged. Biomedical studies are compared using formulation-specific descriptors such as nanosheet size/thickness, coating chemistry, dose, administration route, degradation, biodistribution, clearance, and organ-specific toxicity. Across all three areas, the same question is asked: does interface engineering solve the original limitation, or merely move it to another part of the system?

The scope is deliberately centered on black phosphorus and few-layer phosphorene nanosheets rather than the broader family of phosphorus allotropes. Red phosphorus is mentioned only when it offers a useful comparison or when phosphorus-based electrode studies help clarify questions such as conductive-matrix design and volume-change management. The goal is to connect recent processing and surface chemistry with quantitative performance in energy storage, catalysis, and biomedical applications, and then to identify the reporting practices that would make results from different laboratories easier to compare. Table 1 summarizes how the present review differs from recent related reviews.

## 2. Fundamental Structure, Surface Chemistry, and Theoretical Basis

### 2.1. Crystal Structure, Thickness, and Anisotropy

The orthorhombic BP lattice is built from puckered phosphorus sheets. In-plane bonding is covalent, whereas adjacent sheets are held together by weaker van der Waals interactions. Exfoliation therefore produces a high-area surface without destroying the basic P-P framework, provided that the mechanical, chemical, or electrochemical driving force is controlled. The armchair and zigzag directions are not equivalent. This anisotropy affects charge transport, phonon propagation, mechanical deformation, and ion diffusion, so average particle size alone is an incomplete descriptor of electrochemical or catalytic behavior.

Thickness is the first important design variable. Reducing bulk BP to a few layers increases the fraction of atoms exposed to the surrounding medium and changes the electronic structure. That can shorten ion-transport distances and improve coupling to catalysts, polymers, or biomolecules. It also accelerates surface reactions. In practice, a batch containing 2–5 layer sheets and a batch described only as “nanoscale BP” should not be expected to age, disperse, or react in the same way. Recent papers increasingly report AFM thickness distributions, TEM lateral dimensions, XPS P-O_x_ fractions, and Raman signatures together; this is a useful shift because no single technique adequately describes a reactive 2D solid.

### 2.2. Defects, Oxidation, and Theoretical Descriptors

Defects are the second variable. HRTEM work on bipolar-exfoliated phosphorene identified monovacancies, divacancies, adatoms, grain boundaries, and local amorphous domains while the remaining lattice retained orthorhombic order [11]. Density-functional calculations in the same study linked oxygen chemisorption and P-P bond breaking to the observed amorphization [11]. This is an important reminder when interpreting ‘defect engineering.’ A vacancy can create a favorable adsorption site or redistribute charge, but an uncontrolled vacancy population can also be the entry point for oxidation and structural loss.

**Figure 1:**
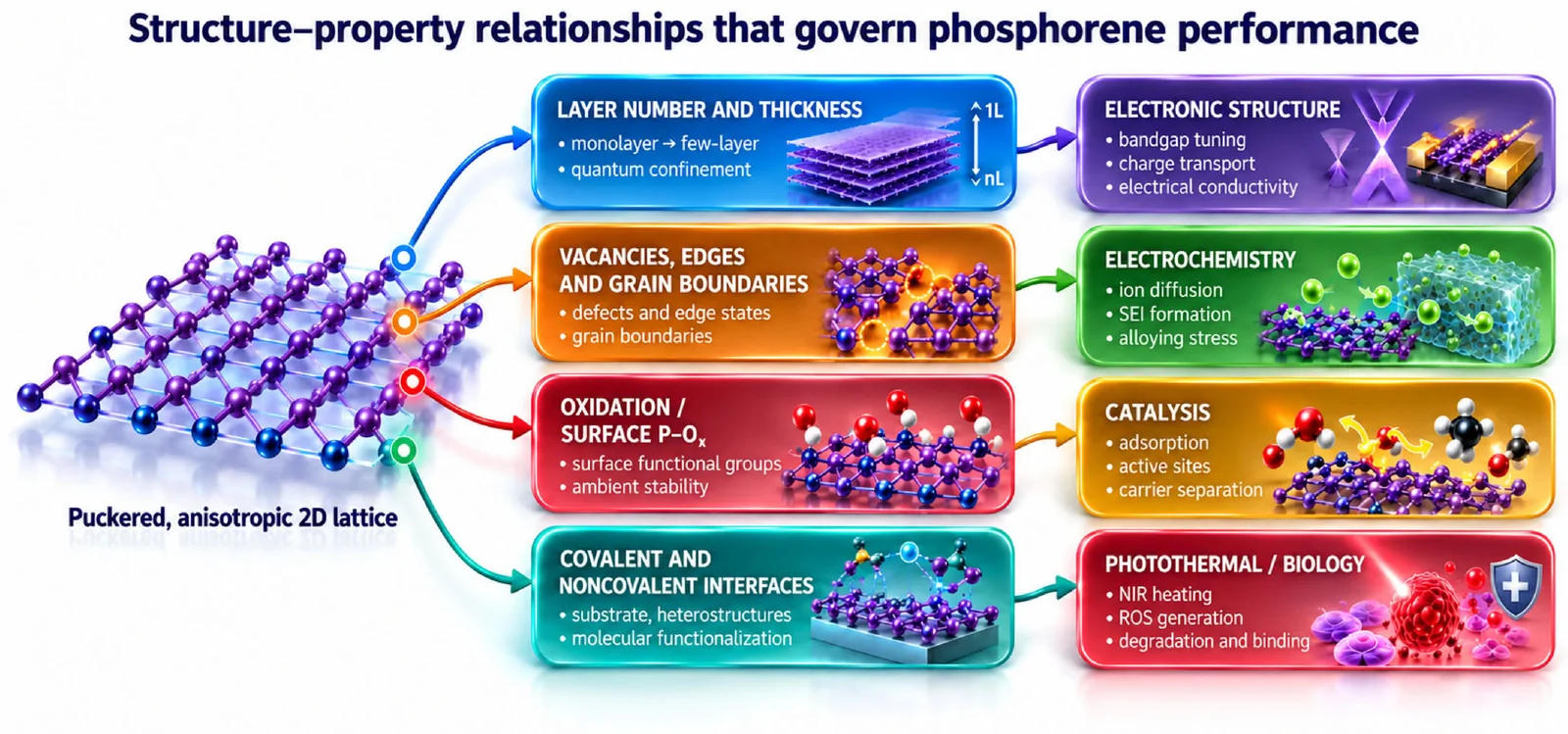
Structure–property map for phosphorene. Thickness, structural defects, oxidation, and interfacial bonding change the electronic, electrochemical, catalytic, and biological response simultaneously; consequently, surface stabilization has to be designed for the intended use rather than applied as a generic post-treatment.

Surface oxidation is not simply a binary distinction between pristine and degraded BP. Phosphorus-oxygen species can passivate some reactive sites, modify hydrophilicity, change electronic transport, and create binding sites for metal species or polymers. The useful state is therefore application dependent. In a battery, excessive P-O_x_ may increase irreversible reactions and impedance; in a composite catalyst, interfacial P-O-M bonding may help stabilize the heterostructure; in biomedicine, controlled degradation to phosphate-containing products may be desirable provided that the dose, kinetics, and organ exposure are understood. Figure 1 summarizes these coupled structure–property relationships.

Representative theoretical results help explain why these structural descriptors matter. The calculated bandgap evolves from roughly 0.3 eV in bulk BP toward values approaching 2 eV in the monolayer/few-layer limit, while the puckered lattice produces strongly direction-dependent effective masses, carrier transport, phonon propagation, and ion migration [1]. For sodium storage, earlier first-principles work identified very low migration barriers along the zigzag direction, consistent with the idea that crystallographic orientation can influence rate capability. Defect calculations and the authors’ HRTEM/DFT study further show that vacancies and oxygen adsorption can lower local bond stability and promote P-P bond breaking and amorphization [11]. Thus, a defect that improves adsorption or ion access at low concentration can become a degradation nucleus when its population or oxidation state is uncontrolled.

Theoretical descriptors should therefore be connected to experimentally measurable quantities. Layer number should be linked to AFM/Raman signatures; defect populations to HRTEM or statistically meaningful spectroscopy; oxidation to quantitative P 2p XPS fitting; and proposed interfacial charge transfer to binding-energy shifts, work-function measurements, or operando spectroscopy. In energy storage, calculated adsorption or diffusion barriers are most persuasive when paired with rate capability and structural evolution. In catalysis, adsorption free energies should be interpreted together with the possibility of surface reconstruction. In biomedicine, calculations of molecular adsorption or degradation energetics are useful only if the final coated formulation is characterized under the relevant biological medium. Table 2 summarizes the principal structure–property descriptors and their minimum supporting evidence.

## 3. Recent Advances in Synthesis, Exfoliation, and Stability Engineering

### 3.1. Liquid-Phase Processing: Improving Size Control Without Ignoring Chemistry

Liquid-phase exfoliation remains attractive because it can process gram-scale feedstock without the small-area limitations of mechanical exfoliation. The recent emphasis, however, is less about proving exfoliation and more about controlling the product distribution. Sarwar et al. optimized NMP-based sonication and reported BP nanosheets with lateral dimensions up to about 5.37 µm, while nanoparticle fractions averaged roughly 68 nm [16]. The work illustrates a recurring tradeoff: aggressive sonication increases exfoliation but also fragments sheets and can generate edge defects. Centrifugation conditions then become part of the synthesis rather than a simple purification step because they determine which size/thickness population is ultimately tested.

The solvent question is also changing. Conventional high-boiling polar aprotic solvents can stabilize dispersions but are inconvenient for biomedical processing and can leave residues that affect electrochemistry. Caponio et al. compared water, Cyrene, and NMP for producing BP nanolayers and showed that safer media can yield dispersions suitable for biological studies [17]. The point is not that water has become universally preferable. Water simultaneously enables convenient processing and accelerates hydrolysis/oxidation unless oxygen exposure, additives, and storage time are managed. A useful synthesis paper should therefore report not only yield and thickness but also the time between exfoliation and characterization, oxygen content, and the chemical state after solvent removal.

For a review focused on 2023–2026, it is also worth separating genuine process advances from refinements of established sonication. Larger lateral size, narrower thickness distributions, benign solvents, and integration with a stabilizing molecule are meaningful improvements because they change downstream performance. By contrast, simply exchanging one organic solvent for another without a clear gain in yield, stability, or application compatibility adds little to the practical picture.

### 3.2. Exfoliation Efficiency, Recovery Yield, and the Centrifugation Bottleneck

For liquid-phase processing, “exfoliation yield” should be separated from the yield of the few-layer fraction that is actually useful. Sonication may detach a large amount of phosphorus from the parent crystal, yet differential centrifugation can leave much of that mass in the sediment as unexfoliated or thick-layer material. Conversely, aggressive sonication can raise the dispersed concentration while reducing lateral size and increasing edge defects. Reporting only the concentration of the final supernatant therefore overstates process efficiency. A useful mass balance is: starting BP mass → dispersed mass after exfoliation → mass retained after size-selection → few-layer mass meeting the target thickness → mass ultimately incorporated into the device or formulation [1].

Existing strategies address this bottleneck in different ways. Cascade centrifugation narrows the thickness and lateral-size distribution but sacrifices recovery. Solvent/surfactant selection improves colloidal stabilization and can reduce reaggregation, although residual molecules may later impede electrochemistry. Intercalation-assisted electrochemical methods weaken the interlayer interaction more directly and can shorten processing time, while bipolar exfoliation can couple delamination with electrophoretic capture on a current collector and thereby avoid a separate collection/redispersion step [1,18]. High-shear and wet-jet approaches reported in the broader BP literature demonstrate that very high apparent exfoliation yields are possible, but those values should not be equated automatically with monolayer/few-layer yield or with device-ready recovery.

For scale-up, the most informative metric is therefore not a single yield number but a yield-quality map. At minimum, studies should report starting mass, recovered dispersed mass, centrifugation sequence, concentration, thickness and lateral-size distributions, P-O_x_ fraction, and usable device/formulation yield. This distinction also helps explain why two methods with similar dispersion concentrations can produce very different electrochemical or biological outcomes.

### 3.3. Electrochemical Exfoliation and One-Pot Functionalization

Electrochemical routes offer two advantages that are difficult to obtain simultaneously by sonication: an electric field can drive specific intercalating ions, and surface chemistry can be coupled directly to the exfoliation step. Kuchkaev et al. used cathodic exfoliation in the presence of iodomethane to generate methyl radicals and form P-C bonds during nanosheet production [19]. Solid-state 31P NMR indicated a functionalization degree near 9.7%, demonstrating that exfoliation and covalent modification do not have to be sequential operations. This type of one-pot chemistry is attractive because a freshly created BP surface is protected before prolonged exposure to air or water.

Wu et al. followed a related process-integration logic using electrochemical exfoliation together with AlO_x_ deposition [20]. The capping layer improved ambient and thermal stability, and the process also yielded a twisted phosphorene structure with a reported twist angle of 9.86°. The energy-storage implications of the twist itself remain to be established, but the study is valuable because it treats passivation as part of synthesis rather than as a later correction to an unstable product.

Bipolar electrochemistry provides another route to integrate material production and electrode fabrication. In the earlier demonstration by Rabiei Baboukani et al., a wireless BP bipolar electrode was exfoliated in deionized water and phosphorene was deposited on a positive feeding electrode, producing a binder-free material that retained capacitive response for at least 40,000 cycles [18]. Recent work from the same line of research extended the concept to reduction-driven deposition on the negative feeding electrode in deionized water, again without organic solvent, binder, or conductive additive [21]. The reported areal capacitance was about 11 mF cm^−2^ at 2 mV s^−1^, and the negative-electrode deposit outperformed the earlier positive-electrode configuration under comparable conditions [21]. The underlying bipolar-exfoliation concept was also protected as a U.S. patent, reflecting its intended relevance to scalable processing [22]. The broader significance is the ability to choose where exfoliated nanosheets land and to remove several non-active components from the device architecture.

Direct deposition also forces a useful question that is sometimes hidden in powder-based studies: which fraction of the exfoliated material is actually electrically connected to the current collector? In conventional slurry electrodes, yield is usually measured before mixing with binder and carbon. In direct-deposition methods, exfoliation yield, capture efficiency, adhesion, and electrochemically accessible loading are coupled. Future scale-up studies should report all four.

### 3.4. Passivation That Can Be Tuned—Or Removed

Recent passivation work has moved beyond thick encapsulation layers toward molecular and chemically reversible protection. Eisenkolb et al. examined a library of perylene diimides and found that water-soluble amphiphilic derivatives with charged head groups can adsorb strongly enough to protect BP flakes in aqueous media for more than 30 days [23]. Mu et al. used a self-assembled ferrocene layer; flakes thinner than 10 nm showed minimal degradation over 25 days, and calculations indicated that adsorption also modified the band structure [24]. These studies show why a stability number should never be quoted without describing the surrounding chemistry: the protecting layer can be electronically active.

Guo et al. introduced a particularly useful concept in 2025: a protective layer that is intentionally removable [25]. Tannic acid first forms a covalent modification that reduces the reactivity of surface lone pairs, followed by Fe(III)-tannic-acid chelation to form a shielding layer. The protected BP was reported to remain stable for more than five months under ambient conditions, while weak-acid ultrasonication could deprotect the surface [25]. For applications in which BP must survive storage but later expose active sites, reversible protection is more attractive than permanent encapsulation.

The field should nevertheless avoid using “stable” as a single universal metric. Optical appearance, Raman peak retention, AFM roughness, XPS P-O_x_ fraction, conductivity, electrochemical impedance, catalytic overpotential, and biological function probe different failure modes. A molecular layer may preserve morphology while changing carrier transport; an encapsulation layer may suppress oxidation while blocking ion access; and a biomedical coating may improve storage stability while changing protein adsorption and organ exposure. Stability should therefore be reported as a time-dependent property under a defined atmosphere or medium and against an application-specific endpoint. Recent stability-focused reviews reach the same conclusion: physical encapsulation, covalent/noncovalent functionalization, surface coordination, and edge protection are not interchangeable because they trade surface accessibility against chemical protection [8].

### 3.5. Bulk BP Preparation and Scale-Up

Scalable phosphorene still depends on scalable, consistent bulk BP. This upstream issue receives less attention than exfoliation but can dominate cost and reproducibility. Zhao et al. reported a vacancy-rich Sn-P-I catalyst for BP preparation and described a short high-temperature conversion route with a yield above 95% under optimized conditions [26]. Such work matters because nanosheet quality cannot exceed the quality and impurity control of the starting crystal. Trace catalyst residues that are tolerable in an electronic material may become problematic in catalysis or biomedicine, where even low metal contamination can distort activity or toxicology.

**Figure 2:**
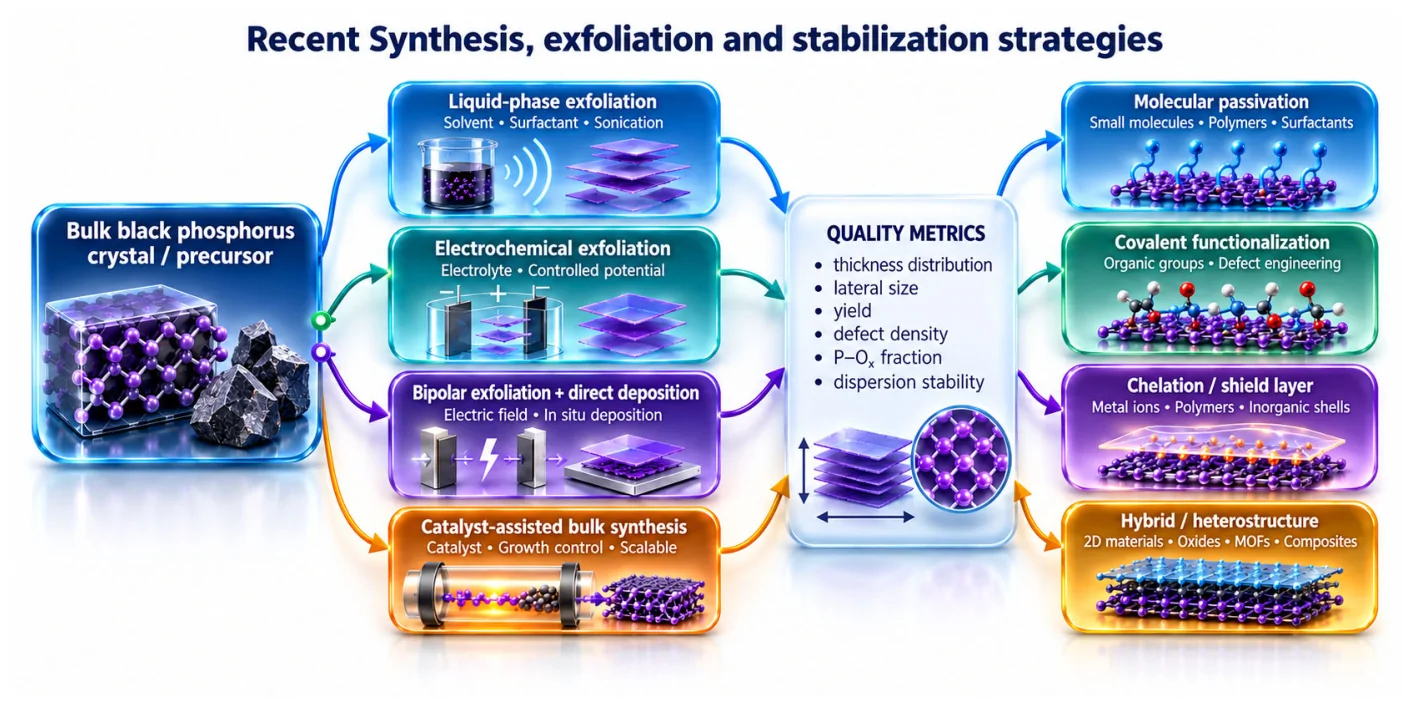
Recent synthesis and stabilization strategies. The emerging objective is not merely thin-sheet production, but reproducible control of nanosheet dimensions, defects, surface oxidation, deposition location, and protective chemistry. Arrows trace the conceptual progression from bulk BP and processing routes through product-quality assessment to stabilization strategies; they do not denote a single chemical reaction.

For future comparisons, bulk synthesis papers should provide phase purity, residual catalyst chemistry, crystal size distribution, and a downstream exfoliation test. Exfoliation papers should state the BP source and lot, because differences in crystallinity, impurity content, and crystal dimensions can easily be mistaken for differences in exfoliation chemistry. Figure 2 summarizes the way synthesis, quality metrics, and protection strategies are now becoming inseparable.

### 3.6. Characterization Standards for a Reactive 2D Material

A recurring problem in the phosphorene literature is that the material described in the synthesis section is not always the material that reaches the application test. BP can change during centrifugation, washing, drying, transfer to water, electrode fabrication, storage, or laser illumination. A characterization package taken immediately after exfoliation is therefore only a starting point. For a reactive 2D material, the chemically relevant specimen is the one present at the moment of electrochemical cycling, catalytic operation, or biological exposure.

Raman spectroscopy remains useful for confirming the BP lattice and tracking thickness- or strain-related changes, but Raman alone does not quantify oxidation. XPS should be used to report the relative P-P and P-O_x_ contributions, and the fitting procedure should be stated clearly because background choice and peak constraints can materially change the reported oxidized fraction. AFM provides thickness and roughness, but a few selected flakes are not a distribution. At least tens of flakes from independent regions should be measured when thickness is being linked to performance. TEM/HRTEM is indispensable for defects and crystallinity, yet the electron beam can itself damage phosphorene. Low-dose imaging and explicit reporting of acquisition conditions are therefore important.

For dispersions, concentration should be measured independently rather than inferred from color. ICP-OES/ICP-MS, gravimetry after complete solvent removal, or a calibrated optical extinction coefficient can be used depending on the system. Dynamic light scattering can help track aggregation but should not replace direct lateral-size microscopy for plate-like particles. Zeta potential is particularly relevant for biomedical and aqueous catalytic formulations because it helps explain colloidal lifetime and protein or ion adsorption.

Surface-state history should be part of the sample name or metadata. A practical reporting format would include: BP source and purity; exfoliation date; solvent/electrolyte; oxygen-control method; centrifugation sequence; storage atmosphere and temperature; elapsed time before characterization; thickness and lateral-size distributions; P-O_x_ fraction; and any coating or functionalization stoichiometry. This information may look procedural, but it is often the difference between a reproducible phosphorene study and one that cannot be compared across laboratories.

The 2022 defect analysis of bipolar-exfoliated phosphorene is a useful example because HRTEM and first-principles calculations were used together to connect observed vacancy/grain-boundary structures with oxidation-driven amorphization [11]. Recent passivation studies similarly combine AFM aging, XPS/Raman, and calculations [23,24,25]. The field should continue in this direction: performance claims are strongest when morphology, chemistry, and mechanism are measured on the same material state. Representative synthesis and stabilization strategies are compared in Table 3.

## 4. Recent Progress in Energy Storage

### 4.1. The Problem Has Shifted from Capacity to Interface Survival

The theoretical capacity of phosphorus makes BP attractive as an alloying-type anode, but recent work reinforces a point that was already visible in early studies: demonstrating high capacity in a low-loading half-cell is comparatively easy; retaining electrical contact, controlling first-cycle loss, and preserving the interface at practical loading are much harder. The structural anisotropy of BP adds another variable because deformation and ion motion are direction-dependent. Accordingly, the most convincing recent electrodes use covalent bonding, conductive scaffolds, confinement, or direct deposition to preserve electron pathways while maintaining ion access. In this review, gravimetric capacity is therefore treated as only one descriptor. Active-material loading, initial Coulombic efficiency (ICE), areal capacity, electrode density, electrolyte inventory, and full-cell validation are emphasized whenever the original paper reports them [7].

The authors’ earlier phosphorus work provides a useful comparison. A red-phosphorus/sulfurized-polyacrylonitrile (RP-SPAN) composite produced by high-energy milling and electrostatic spray deposition delivered 1605 mAh g^−1^ at low current and 696 mAh g^−1^ at 1 A g^−1^ [27]. A later stability analysis showed nonlinear and time-varying low-frequency behavior with an average capacity loss of about 0.21% per cycle [28]. Although red phosphorus and phosphorene are structurally different, the lesson is common: a conductive, mechanically accommodating matrix can improve kinetics, but a headline capacity does not replace time-domain stability analysis.

**Figure 3:**
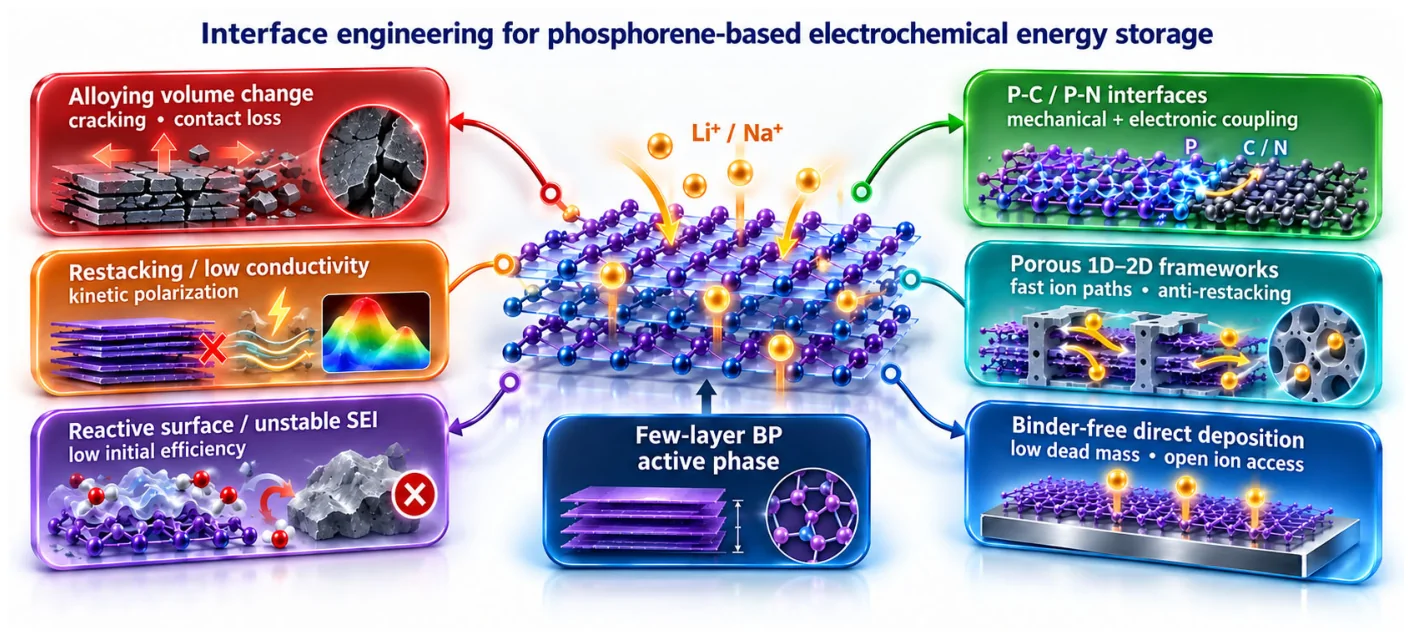
Major failure modes and interface-engineering strategies for phosphorene-based electrochemical energy storage. Practical assessment increasingly requires areal and full-cell metrics in addition to gravimetric capacity.

Figure 3 frames the current BP electrode design around three coupled failure modes: alloying-driven strain, electronic isolation/restacking, and reactive interfaces. Recent composites increasingly target more than one of these at the same time.

### 4.2. Lithium-Ion Batteries: Conductive Networks and Chemically Bonded Matrices

Yuan et al. combined BP with polyaniline and carbon nanotubes to create a three-component anode in which the polymer and CNT network accommodated expansion and improved electrical continuity [29]. The composite delivered an initial discharge capacity above 2000 mAh g^−1^ and retained roughly 1150 mAh g^−1^ after 100 cycles. A separate 2023 BP/graphite/Sn design reported more than 2000 mAh g^−1^ after 50 cycles at a moderate current and still hundreds of mAh g^−1^ at 2 A g^−1^ [30]. These results are encouraging, but the more important trend is architectural: the phosphorus phase is increasingly treated as a high-capacity guest that needs a mechanically persistent conductive skeleton.

The next step for LIB studies is to move from optimized half-cell data to metrics that expose practical penalties. Many BP composites contain a large fraction of carbon, polymer, or inactive scaffold; reporting capacity per mass of phosphorus can therefore exaggerate electrode-level energy density. Electrode thickness, total active loading, initial Coulombic efficiency, electrolyte volume, and capacity normalized to the complete composite should be reported together. Prelithiation requirements are especially important because high first-cycle loss is difficult to hide in a full cell with a limited cathode lithium inventory.

### 4.3. Sodium-Ion Batteries: Covalent Anchoring and Multiscale Conductive Scaffolds

Sodium-ion batteries remain one of the strongest use cases for BP because the high phosphorus capacity can offset the lower cell voltage and energy density of sodium chemistries. The challenge is severe volume change together with slower transport and unstable interfaces. Ai et al. used a black-phosphorus/hard-carbon nanocomposite containing stable P-C and P-O-C bonding [12]. The optimized material delivered a high initial capacity and retained more than 1800 mAh g^−1^ after 50 cycles at 0.1 A g^−1^. The interface was not merely a conductive contact; chemical bonding helped maintain the composite during repeated sodiation/desodiation.

A 2025 BP/Ti_3_C_2_T_x_ MXene/CNT composite extended this concept to a multicomponent scaffold [13]. Stable P-O-Ti bonds were associated with a reversible capacity of 630.7 mAh g^−1^ after 1400 cycles at 2 A g^−1^, and 281.1 mAh g^−1^ was retained after 1000 cycles at 10 A g^−1^ [13]. These values are more informative than an isolated low-rate capacity because they demonstrate long-term interfacial survival under demanding kinetics. The architecture also illustrates a useful separation of functions: the 2D MXene supplies a conductive, chemically bonded interface, CNTs maintain long-range electronic connectivity, and BP supplies high-capacity sodium storage. A full-cell demonstration with an NFPP cathode was also reported, although the literature still lacks the electrode-density, N/P-balance, and energy-density accounting needed for a cell-level comparison.

For sodium storage, future studies should quantify the fraction of capacity that is diffusion controlled versus surface/capacitive, but kinetic fitting should not be used as a substitute for direct structural evidence. Operando XRD, Raman, pair-distribution analysis, or electron microscopy can show whether the designed P-C or P-O-M interface survives the sodiation window or reconstructs after the first cycle.

### 4.4. Lithium-Sulfur Batteries: Phosphorene as a Catalytic Interface Rather than a Bulk Anode

The role of BP in Li-S batteries is conceptually different. Here, few-layer BP is used as a polar, catalytic interface that adsorbs lithium polysulfides and accelerates their conversion rather than as the main capacity-bearing phase. Zhou et al. covalently bonded few-layer BP to nitrogen-doped carbon-coated CNTs, creating a P-N heterointerface [14]. Under electrolyte-lean conditions the catalyst supported an areal capacity of 7.69 mAh cm^−2^ at a sulfur loading of 8.74 mg cm^−2^ and an electrolyte/sulfur ratio of 5.7 µL mg^−1^. In pouch-cell testing at lower electrolyte inventory, the system retained 70% of capacity after 200 cycles [14]. These are useful metrics because they approach the constraints that matter in high-energy Li-S cells.

Chen et al. used BP nanosheets on CNTs as a separator modifier to improve polysulfide conversion [31]. A 2025 red/black-phosphorus heterostructure anchored on graphene similarly combined polysulfide adsorption with catalytic conversion and conductive transport [32]. More recently, Li et al. dispersed a small amount of black phosphorene into a composite polymer solid electrolyte membrane [33]. With 0.3 wt% BP, the membrane showed an electrochemical stability window of 5.37 V and ionic conductivity of 1.3 × 10^−3^ S cm^−1^ at 60 °C; the resulting solid-state Li-S cell delivered 1322 mAh g^−1^ initially and retained 453 mAh g^−1^ after more than 100 cycles at 0.05 C [33]. These examples show that BP can influence sulfur chemistry even when it is not a major mass fraction of the cell.

The strongest Li-S papers now report the quantities that dominate practical sulfur-cell energy density: sulfur loading, electrolyte/sulfur (E/S) ratio, areal capacity, catalyst mass, and pouch-cell or solid-state behavior. For example, the c-FBP-NC host delivered 7.69 mAh cm^−2^ at 8.74 mg cm^−2^ sulfur and an E/S ratio of 5.7 µL mg^−1^; its pouch cell used 4 mg cm^−2^ sulfur at E/S = 3.5 µL mg^−1^ and retained about 70% of capacity after 200 cycles [14]. These boundary conditions are more informative than a high specific capacity obtained from dilute electrolyte or low sulfur loading. A catalyst that looks excellent at ~1 mg cm^−2^ sulfur and large electrolyte excess may offer little advantage at the electrode level.

### 4.5. Supercapacitors and Binder-Free Phosphorene Electrodes

Supercapacitors place different demands on BP. Large accessible surface area and fast ion response are useful, but restacking and oxidation can reduce the active area quickly. Xin et al. chemically bridged BP with conductive C_3_N_4_ through P-C bonds [15]. The flexible device delivered a volumetric capacitance of 42.1 F cm^−3^, an energy density of 5.85 mWh cm^−3^, and no measurable capacitance loss over 30,000 cycles [15]. The result is a good example of interfacial chemistry serving both mechanical and electrical roles.

Halder et al. reported a scalable wet-chemical/hydrothermal route to few-layer phosphorene and used it in an all-solid asymmetric supercapacitor [34]. Other 2023–2025 studies have combined BP with heteroatom-doped carbon microspheres, MoS2, or conductive carbon nitride to improve conductivity and suppress restacking [35,36]. The key comparison, however, is not just F g^−1^. For thin 2D electrodes, volumetric and areal capacitance, total device thickness, electrolyte mass, and bending stability are often more useful than gravimetric values.

Bipolar direct deposition is attractive in this context because it can eliminate polymer binder and added carbon. The 2019 positive-feeding-electrode study established high cycling stability [18], while the 2026 negative-feeding-electrode study reported substantially higher areal capacitance under comparable testing [21]. This polarity effect deserves further mechanistic study. It may reflect differences in local reduction/oxidation chemistry, deposition morphology, surface oxidation, and adhesion. Importantly, direct deposition also provides a route to pattern or localize BP without collecting, drying, redispersing, and casting a powder.

### 4.6. What Has Actually Changed Since the 2021 Energy-Storage Picture?

Comparing the current literature with the state summarized in 2021 [1] is instructive. The fundamental storage mechanism has not been reinvented: phosphorus still offers high alloying capacity and still experiences large structural change. What has changed is the level at which the interface is engineered. Earlier composites often used graphene or CNTs mainly as conductive additives and mechanical buffers. Recent work is more likely to establish a chemically bonded P-C, P-N, or P-O-M interface and then use spectroscopy or calculations to explain how that bond changes charge transfer and structural retention [12,13,14,15].

A second change is the quality of testing in the most convincing studies. Electrolyte-lean Li-S cells, high sulfur loadings, long-cycle sodium-ion data at ampere-per-gram current densities, and binder-free areal capacitance are closer to application constraints than the low-loading half-cell demonstrations that dominated the early literature. This does not mean that practical BP batteries have arrived. It means that the field is beginning to expose the penalties—inactive scaffold mass, first-cycle loss, electrolyte demand, and electrode density—that determine whether phosphorene can contribute at the cell level.

A third change is the role of BP itself. It is no longer used only as an anode. In Li-S systems, a small amount of few-layer BP can act as a catalytic/polar interface for polysulfide conversion [14,31,32,33]. In polymer electrolytes, BP can modify interfacial contact and ion transport [33]. In supercapacitors, direct deposition can turn exfoliation into the electrode-fabrication step [21]. These uses may ultimately be more scalable than asking BP to supply most of the electrode mass.

The remaining gap is electrode manufacturing. Few studies report slurry rheology, calendaring response, dry-electrode compatibility, electrode density, or large-area coating uniformity. Direct-deposition routes avoid some of these problems but introduce others, including capture efficiency and loading uniformity. A realistic next phase should compare BP architectures using the same areal loading and electrode thickness and then follow them into full cells rather than optimizing each material in its own favorable test window. Table 4 consolidates representative electrode-level performance and reporting gaps.

## 5. Catalysis and Energy Conversion

### 5.1. Phosphorene Is Often a Chemically Active Support, Not an Inert 2D Substrate

Catalysis makes the dual nature of BP particularly obvious. Lone-pair electrons can bind metals, intermediates, and adsorbates, which helps create active sites; the same electron density makes BP vulnerable under oxidative potentials. A good catalyst design therefore has to tune the P electronic structure without simply converting the nanosheet into an uncontrolled oxide or phosphide. Recent studies increasingly use covalent metal-P bonds, heterostructures, and vacancies to redistribute charge and stabilize selected intermediates.

Zhai et al. functionalized BP during electrochemical exfoliation with only 1.5 wt% Ni [37]. The resulting Ni-P bonding redistributed charge and produced bifunctional alkaline activity: overpotentials of 136 mV for HER and 230 mV for OER at 10 mA cm^−2^, together with roughly 50 h of overall-water-splitting stability [37]. The result is notable because the metal loading is low and the bonding environment—not simply nanoparticle loading—was treated as the design variable. Fang et al. used N-doped CoP on ammoniated BP and reported HER overpotentials of 90 mV at 10 mA cm^−2^ and 246 mV at 200 mA cm^−2^ in alkaline electrolyte [38]. Both examples support the same mechanism-level conclusion: BP is useful when it participates in interfacial electron redistribution rather than when it acts only as a high-area support.

### 5.2. Oxygen Evolution: Stability Under Deliberately Oxidative Conditions

OER is a stringent test for phosphorene because the catalyst is operated at potentials that favor oxidation. Jiang et al. coupled nickel cobaltite with BP and used interfacial bonding to modulate the electronic structure, obtaining an overpotential near 197 mV at 10 mA cm^−2^ [39]. Mao et al. subsequently combined oxygen-deficient NiFe_2_O_4_ with exfoliated BP; P-Ni and P-O-M bonding was proposed to stabilize the interface and the heterostructure reached 243 mV at 10 mA cm^−2^ with a Tafel slope of 39 mV dec^−1^ and nearly 100 h of testing [40]. A related 2024 study on P-O-bond-regulated cobalt oxide/BP interfaces reached 269 mV at 10 mA cm^−2^ [41].

These values are competitive, but the more important scientific issue is whether the synthesized BP-containing interface is also the operating active state. Under OER bias, transition-metal surfaces commonly reconstruct toward oxyhydroxide-like phases, while BP can oxidize, dissolve, or form P-O-M environments. The catalytic material should therefore be viewed as a time-dependent sequence—precatalyst → reconstructed interface → steady-state active phase—rather than as a static structure. Ex situ XPS after operation can show that phosphorus remains, but it cannot by itself prove that the original P-M bond is the turnover site. Operando Raman or X-ray absorption, time-resolved XPS where feasible, isotope labeling, phosphorus dissolution/mass balance, and post-test cross-sectional microscopy are stronger evidence. A durable current trace with substantial chemical reconstruction should be described as a stable performance of a reconstructed catalyst, not necessarily the stability of pristine phosphorene.

### 5.3. Photocatalytic Hydrogen Production: Heterojunctions and Charge Separation

The broad optical absorption of BP makes it attractive for photocatalysis, but rapid carrier recombination and chemical instability limit pristine nanosheets. Heterojunctions are therefore the dominant recent strategy. A 2D/2D BP/TpPa-1 covalent-organic-framework p-n junction reported in 2023 produced hydrogen at 456.7 µmol h^−1^ g^−1^ without a noble-metal cocatalyst, about 13 times the COF and 24 times the BP component under the reported conditions [42]. The gain was attributed to more efficient separation and transport across the p-n interface.

Provinciali et al. used BP to promote TiO_2_ and then added CoP, reaching a much higher hydrogen-evolution rate than pristine TiO_2_ [43]. Phase engineering can also be used within phosphorus itself: a violet/black-phosphorus heterostructure extended visible-light response and increased hydrogen production relative to its individual components [44]. These papers show that the useful role of BP in photocatalysis is frequently as a light-absorbing, charge-separating component of a junction rather than as a standalone catalyst.

### 5.4. CO_2_ Reduction: From Vacancy Calculations to Engineered Heterointerfaces

CO_2_ reduction is less mature but scientifically useful because it makes selectivity and intermediate binding explicit. Fan et al. used first-principles calculations to study monovacancy BP and predicted that the local electrostatic potential gradient could promote charge separation and facilitate CO-CO coupling toward acetaldehyde [45]. The work is theoretical, but it highlights why controlled defect density could be beneficial if the defect population can be made and stabilized experimentally.

He et al. designed a dual-atom P-Co-Dy charge-transfer bridge on BP for photocatalytic CO_2_ reduction [46], while Duan et al. reported an NH_2_-UiO-66/BP heterojunction in 2026 with an aerogel-inspired interfacial architecture for CO_2_-to-CO conversion [47]. The direction is consistent across these studies: selectivity improves when the BP interface is used to control the electronic environment of adsorbed intermediates. The open challenge is to normalize activity to a defensible number of active sites and to show that the site population is stable under reaction conditions.

### 5.5. Comparing Catalytic Performance Without Losing the Chemistry

The phosphorene catalysis literature would benefit from tighter normalization. HER and OER overpotentials at 10 mA cm^−2^ are useful screening metrics, but they conflate active-site density, electrical contact, catalyst loading, roughness, and intrinsic activity. Reporting mass activity, electrochemically active surface area, turnover frequency when defensible, and metal loading by ICP can reveal whether a performance gain is caused by more sites or better sites. For low-metal BP catalysts such as Ni-BP, this distinction is especially important because the claim is not simply high activity; it is high activity with a small metal inventory [37].

Photocatalytic comparisons are even more sensitive to reactor conditions. The H_2_-evolution rates quoted above are therefore examples of performance within each original study and should not be interpreted as a direct ranking among catalysts. Meaningful cross-study comparison requires incident photon flux or calibrated light intensity, illuminated area, spectral distribution, catalyst loading/concentration, reactor geometry, temperature, sacrificial-agent identity and concentration, and apparent quantum yield at defined wavelengths. A rate expressed only in µmol h^−1^ g^−1^ can change substantially with optical density and light path length. Where these parameters are not reported, the defensible comparison is mechanistic—e.g., whether a heterojunction improves charge separation relative to its internal controls—rather than numerical ranking. CO_2_-reduction studies require the same discipline plus carbon balance and isotope controls to verify product origin.

Stability should be chemical, not only electrochemical or photochemical. A flat current-time trace does not prove that BP remains BP. Phosphorus may dissolve, oxidize, or transform while another phase maintains activity. Post-test XPS/XRD is useful, but operando methods are more persuasive when reconstruction is expected. Measuring phosphorus in the electrolyte after long operation would add a simple mass-balance check that is rarely reported.

**Figure 4:**
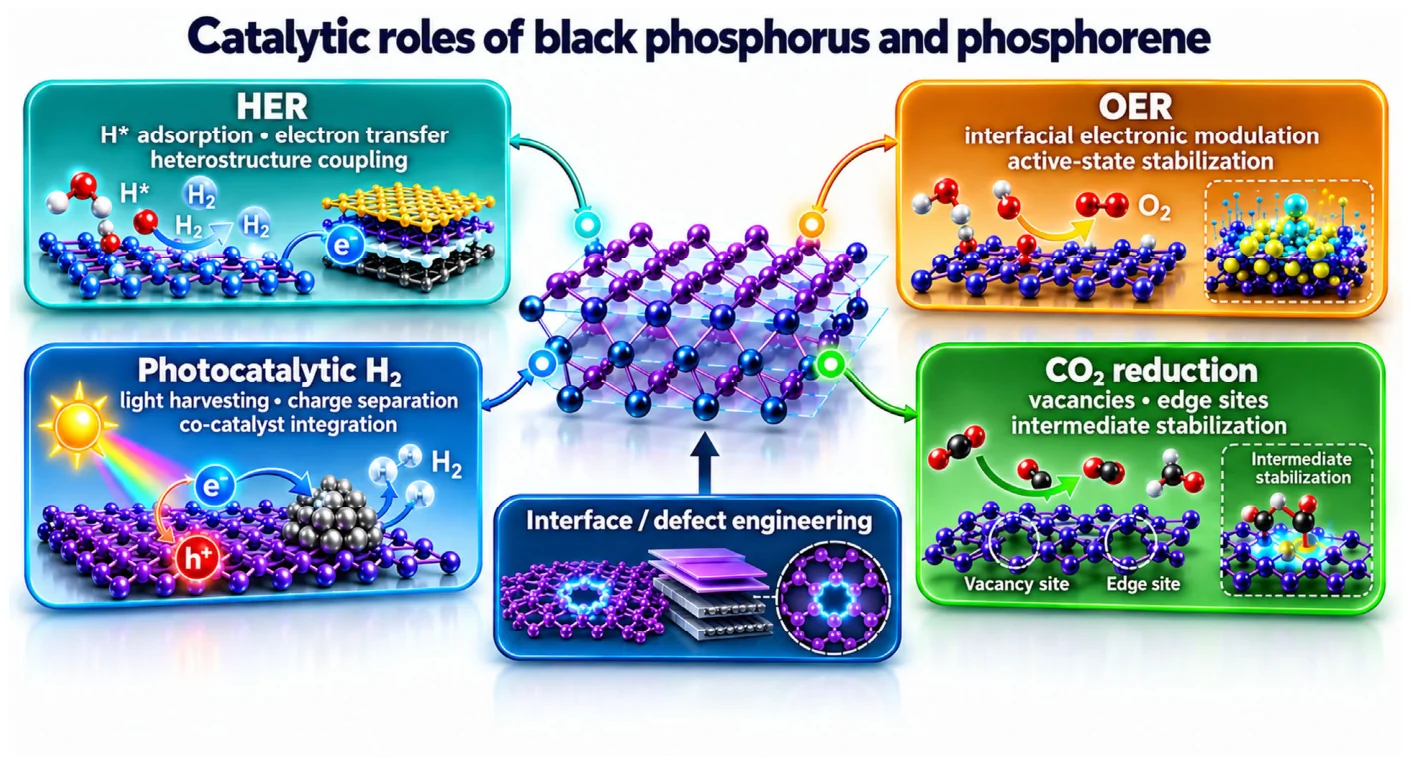
Four current catalytic roles of phosphorene. Interface and defect engineering can improve adsorption and charge separation, but the chemically active state must be verified under operating conditions because BP can oxidize or reconstruct. The electron labels use the true minus sign (e^−^), while mechanistic arrows depict conceptual pathways rather than stoichiometrically complete reactions.

These reporting improvements are not administrative details. They determine whether BP is truly functioning as a durable catalytic phase, an electronic promoter that survives, or a sacrificial precursor that creates a different active surface. Each role can be useful, but they imply different design rules and different prospects for scale-up. Figure 4 illustrates these four interfacial catalytic roles. Table 5 compares representative systems and the evidence needed to establish their operating active states.

## 6. Biomedical Applications

### 6.1. Why Phosphorene Remains Attractive for Medicine

Biomedical phosphorene research is driven by a combination that is difficult to reproduce with many inorganic 2D materials: a high surface area for cargo adsorption, strong near-infrared absorption for photothermal therapy, the ability to participate in reactive-oxygen chemistry, and a phosphorus-based degradation pathway. Recent reviews have catalogued these advantages in cancer therapy, drug delivery, bioimaging, and tissue engineering [5,6]. The important shift in the 2023–2026 literature is that BP is increasingly embedded in multicomponent systems—membranes, hydrogels, niosomes, cell-membrane coatings, or growth-factor scaffolds—rather than administered as an unmodified nanosheet.

This design trend is sensible. A bare BP nanosheet has little control over colloidal stability, protein corona formation, target-cell recognition, or residence time. Surface coatings can improve all of these properties, but they also change the biological identity of the particle. The pharmacology of a polydopamine-coated BP carrier or a cell-membrane-camouflaged BP platform cannot be inferred from the behavior of bare BP. For translation, coating composition and stability should therefore be treated as part of the active product, not as formulation details.

### 6.2. Drug Delivery and Multimodal Cancer Therapy

NIR-responsive delivery remains one of the most active areas because BP itself provides a heat source while the surface can carry therapeutic molecules. Xiang et al. used folic-acid-terminated BP nanosheets for targeted gene delivery and photothermal treatment of hepatocellular carcinoma [48]. The system combined receptor targeting with BP-mediated heating and nucleic-acid delivery, illustrating the appeal of using one nanosheet for multiple functions. Qin et al. went further with a tumor-cell-membrane-camouflaged BP nanocomplex containing evodiamine [49]. The membrane contributed immune evasion and tumor targeting, while BP supported photothermal treatment and the drug component contributed chemotherapy/immunomodulation.

Kumar et al. integrated BP nanosheets into niosomes co-loaded with indocyanine green and doxorubicin [50]. Drug loading exceeded 90%, and 808 nm irradiation provided photothermal/photodynamic effects together with pH- and light-responsive release. This type of combinatorial platform is powerful experimentally, but it also complicates interpretation. If tumor regression improves, it may be difficult to assign the gain to BP heating, ICG photochemistry, doxorubicin release, altered biodistribution, or a combination of all four. Factorial controls and dose-equivalent comparisons are therefore essential in future studies.

Recent cancer-focused reviews have rightly emphasized therapeutic performance [5], but manufacturing questions deserve equal attention: BP thickness distribution, oxidation state at dosing, sterilization, endotoxin, residual solvent, coating stoichiometry, and NIR power density should be reported in a way that allows another group to reproduce the formulation. Without those details, an apparently precise nanoplatform can be chemically different by the time it reaches an animal study.

### 6.3. Crossing Biological Barriers and Treating Non-Cancer Disease

BP’s photothermal behavior is also being used to overcome biological barriers. Yin et al. developed polydopamine-modified BP nanosheets carrying pterostilbene for ischemic stroke treatment [51]. The PDA layer improved stability, while NIR heating was used to enhance blood–brain-barrier transport and trigger drug release. The study illustrates an important advantage of BP: the physical stimulus and the carrier are the same structure. It also raises a safety question, because a platform intentionally designed to alter barrier permeability must be assessed for off-target tissue exposure.

In 2026, He et al. used a more elaborate biomimetic strategy for nephrocalcinosis [52]. DNase I was loaded on BP nanosheets and the construct was coated with a neutrophil membrane modified for targeting. The system scavenged reactive oxygen species, reduced neutrophil extracellular traps, and protected against calcium-oxalate-associated kidney injury. This is a good example of BP acting less as a photothermal agent and more as a redox-active nanomaterial integrated into a targeted biological carrier.

### 6.4. Tissue Regeneration: BP as an Ion Source, Reinforcing Phase, and Photothermal Component

Tissue-regeneration studies exploit different features of BP from cancer therapy. In a vascularized bone-regeneration system, Miao et al. incorporated VEGF-decorated BP nanosheets into a dynamic DNA hydrogel and combined it with a 3D-printed scaffold [53]. The nanosheets reinforced the hydrogel and helped sustain protein delivery; the construct promoted angiogenic and osteogenic responses in a critical-size rat cranial defect model. The work is important because BP is not just an additive. It influences mechanics, protein binding, and the local chemical environment.

Recent bone-regeneration studies continue this multifunctional approach. Li et al. incorporated BP nanosheets into mesenchymal-stem-cell spheroid systems to accelerate bone formation [54], while Wang et al. integrated Zn-doped BP nanosheets into a Janus hydrogel for vascularized bone regeneration [55]. The rationale is that phosphorus-containing degradation products can support mineralization, while metal doping or biological cell organization adds angiogenic and immunomodulatory functions. Care is needed, however, when mechanistic claims are made: enhanced osteogenesis may result from phosphate release, local heating, surface topography, metal ions, or changes in macrophage behavior. Those contributions should be separated where possible.

Wound healing has become another active area. Ge et al. embedded BP nanosheets into a hydrogel-based triboelectric nanogenerator [56]. The patch combined self-powered electrical stimulation with BP-mediated photothermal heating and antibacterial action, accelerating diabetic-wound healing in mice. Li et al. later developed a BP/polydopamine/GelMA system carrying deferoxamine [57]. Mild NIR-triggered heating was coupled with controlled drug release to reduce ROS and inflammation and promote angiogenesis. In both cases, the best feature of BP is not a single material property but its ability to connect an external NIR stimulus with local chemical and biological responses.

Neural repair is emerging as well. Shen et al. fortified BP nanosheets with catalase to improve Schwann-cell responses for nerve repair [58]. This direction is particularly interesting because ROS regulation and phosphorus degradation can be useful without high-temperature photothermal treatment, potentially widening the range of tissues where BP could be used.

### 6.5. Antibacterial Activity: Moving Beyond Photothermal Killing

Many early BP antibacterial systems relied on NIR heating or ROS generation. A 2026 ACS Nano study by Xu et al. introduced a different mechanism [59]. Tannic-acid-armored BP nanosheets were produced by a green ethanol-assisted route and showed light-independent antibacterial activity attributed to curvature-selective membrane interactions. The nanosheets preferentially disrupted bacterial membranes while largely sparing mammalian membranes in the tested models. The platform was effective against multispecies dental biofilms and resistant Staphylococcus aureus infection in mice [59].

This is a useful conceptual advance because it reduces dependence on external irradiation and simultaneously addresses oxidative stability. It also reinforces a point made throughout this review: passivation does not simply ‘protect’ BP. Tannic acid changes dispersion, adhesion, interfacial mechanics, and biological selectivity. In other words, the passivation layer becomes part of the therapeutic mechanism.

### 6.6. Biosafety: Biodegradability Is Not Equivalent to Biological Safety

The most important change in the recent biomedical literature is the growth of explicit toxicology studies. It is no longer adequate to assume that phosphorus-containing degradation products guarantee safety. Dong et al. reported oxidative-stress- and apoptosis-associated effects of BP nanosheets in vascular endothelial cells [60]. A 2024 study showed that BP nanosheets that were tolerated under baseline conditions could become toxic under oxidative stress, with degradation and excess hydroxyl-radical formation contributing to mitochondrial injury [61]. Zhang et al. linked BP nanosheet exposure to hepatocyte pyroptosis through a ROS-NLRP3-GSDMD pathway, while melatonin reduced the effect [62].

The 2026 literature makes the safety question even more specific. Jiang et al. reported that BP nanosheets could cross the blood–testis barrier in mice after repeated exposure and were associated with altered sperm parameters, endocrine disruption, and germ-cell injury [63]. Zhu et al. found that repeated exposure to BP nanomaterials altered hepatic lipid metabolism and mitochondrial function [64]. These studies do not invalidate therapeutic BP. They show that route, dose, formulation, particle size, surface chemistry, and exposure duration matter, and that organ-specific barriers cannot be assumed to block nanosheets.

**Figure 5:**
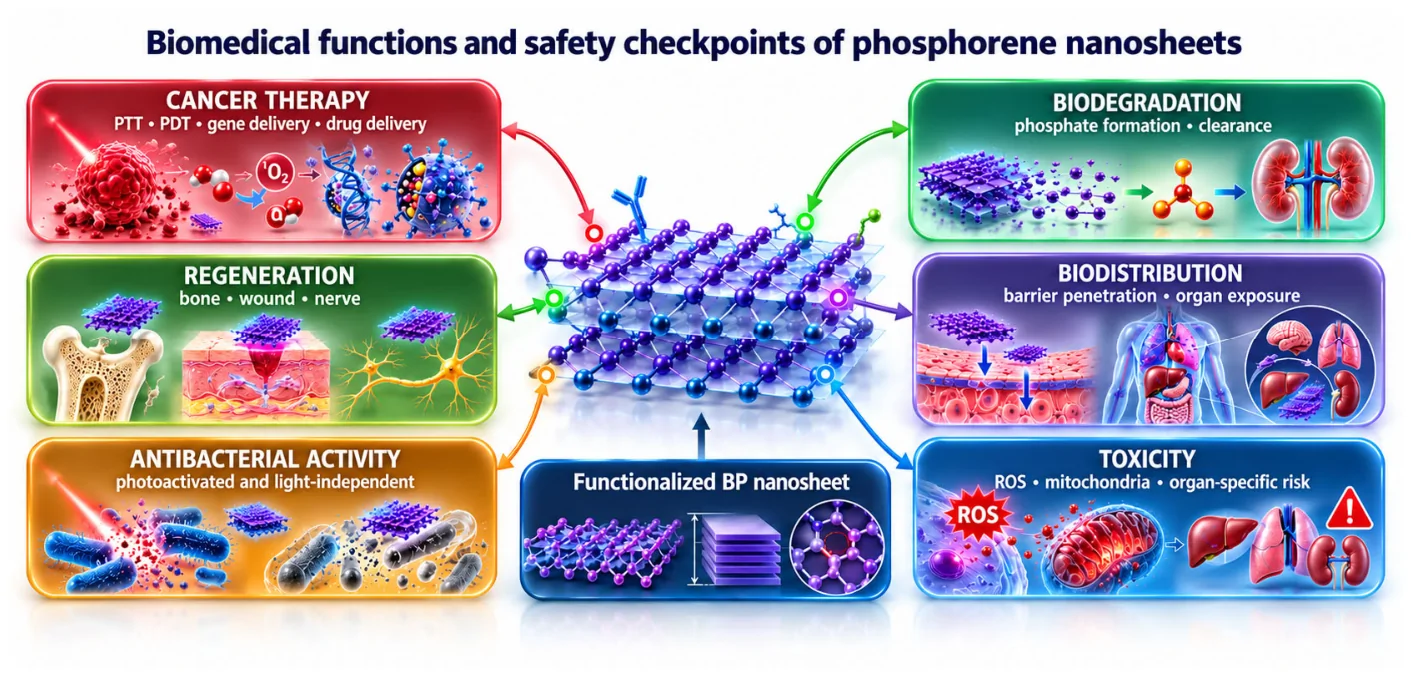
Biomedical functions of phosphorene and the safety questions that accompany them. Coatings, dose, degradation kinetics, barrier penetration, and organ exposure must be evaluated for the final formulation rather than inferred from pristine BP. Arrows connect the functionalized BP nanosheet to potential applications and associated degradation, distribution, and safety checkpoints; they do not imply established clinical efficacy.

The correct translational response is not to label BP safe or unsafe. It is to define a therapeutic window for a specific formulation. A drug-loaded PDA-coated BP nanosheet delivered locally in a hydrogel is a different product from an uncoated BP nanosheet given systemically. Biodistribution and clearance should be measured for the final construct at the intended dose. Degradation kinetics should be characterized in serum or relevant tissue fluid, and phosphorus-containing products should be quantified rather than inferred. Figure 5 summarizes the balance between biomedical function and safety checkpoints.

A systematic safety comparison also makes clear where the evidence is still thin. Size and thickness are often characterized before formulation but not after protein adsorption or storage; administered dose may be reported as total nanocomplex mass rather than BP mass; and fluorescent tracking can persist after a degrading phosphorene carrier has chemically disappeared. For this reason, Table 6 separates formulation, dose/route, biodistribution/clearance evidence, and the reported safety endpoint. “NR” is retained deliberately where the primary study does not provide a parameter that would be required for translation.

### 6.7. Formulation, Biodistribution, and Manufacturing Variables That Need More Attention

The biomedical section of a phosphorene review can easily become a list of increasingly complex nanoplatforms. A more useful comparison asks which formulation variables control exposure. Lateral size affects cellular uptake and filtration; thickness and defect density affect degradation; coating charge changes serum-protein adsorption; polymer or membrane coatings alter circulation time; and aggregation changes the effective dose delivered to small vessels and organs. These variables should be measured after the material has been placed in the relevant biological medium, not only in deionized water.

Biodistribution studies also need stronger mass balance. Fluorescent labels can detach from a degrading BP carrier and produce a signal that no longer tracks phosphorus. Direct phosphorus quantification by ICP-MS/ICP-OES, combined with label-based imaging where needed, would provide a more defensible picture. For coatings containing metals or dyes, each component should be tracked separately. The 2026 blood–testis-barrier and hepatic-metabolism studies [63,64] demonstrate why this matters: a low circulating concentration does not necessarily mean that biologically relevant exposure has ended.

Manufacturing details are equally important. Biomedical-grade BP will need controlled residual solvent, endotoxin limits, sterility, particle-size specifications, oxidation specifications, and a shelf-life definition. Sterilization itself may alter a reactive 2D material; heat, irradiation, filtration, or chemical sterilants should be tested for their effect on P-O_x_ content and coating integrity. Freeze-drying or cryoprotectants may improve storage but can change redispersion and protein binding after reconstitution.

For photothermal systems, thermal dose should be treated as a pharmacological variable. Reporting laser power without the achieved tissue temperature is incomplete. Conversely, reporting only temperature rise without local light intensity makes replication difficult. The most translatable studies will define both, then show that therapeutic effect remains when power density and exposure duration are kept within a clinically plausible range.

These requirements do not make BP uniquely difficult; they are standard problems in nanomedicine. BP simply adds a time-dependent chemical state to them. That is precisely why interface engineering is valuable. A well-designed coating can stabilize the nanosheet long enough for manufacturing and delivery, then allow controlled exposure or degradation at the target site.

## 7. Cross-Application Structure–Property Relationships

Looking across batteries, catalysts, and biomedical systems, the same variables repeatedly appear: nanosheet thickness, lateral size, defect density, oxidation state, covalent bonding, and the accessibility of the basal plane/edges. The applications differ mainly in what they ask those variables to do. Battery electrodes require rapid ion/electron transport and mechanical continuity. Catalysts require selected adsorption energies and charge transfer without destructive reconstruction. Biomedical systems require colloidal stability, stimulus response, controlled degradation, and a predictable biological identity.

This comparison helps resolve an apparent contradiction in the literature. Oxidation is often described as uniformly harmful, yet partially oxidized or P-O-M-containing interfaces can be useful. The issue is control. Uncontrolled oxidation during storage changes the material before testing. Deliberate interfacial oxidation or metal–oxygen–phosphorus bonding can stabilize a composite or tune adsorption. Researchers should therefore report oxidation quantitatively and distinguish native degradation from intentional chemical modification.

The same logic applies to defects. A high vacancy density may be undesirable for charge transport in electronics but beneficial for adsorption or catalytic activation. In batteries, defects can increase electrolyte reactivity and SEI formation even as they increase ion-accessible sites. In biomedical systems, defect-rich sheets may degrade faster and produce more ROS. ‘More defects’ is therefore not a general optimization target. The correct target is a defect population matched to the desired reaction and operating environment.

Covalent interfaces are one of the clearest cross-cutting successes. P-C bonding stabilizes several battery and supercapacitor composites; P-N and P-M bonding can tune catalytic electronic structure; polymer or biomolecule anchoring stabilizes biomedical dispersions. But strong bonding can also block surface sites or slow degradation. This tradeoff is why interfacial chemistry should be designed from the application backward rather than chosen solely for maximum shelf stability.

Finally, measurement conditions must follow the same discipline. A BP battery electrode tested at sub-milligram loading, a photocatalyst illuminated with poorly defined power density, and a biomedical nanosheet tested at a concentration far above a realistic exposure all create impressive but hard-to-translate numbers. Cross-application maturity will come from realistic boundary conditions as much as from higher intrinsic performance.

### Interface Engineering as the Common Language of the Field

Seen from this perspective, energy, catalytic, and biomedical phosphorene studies are not separate branches. They are different tests of interfacial lifetime. A battery asks whether an interface survives repeated changes in chemical potential and volume. A catalyst asks whether the surface can exchange charge and bind intermediates without reconstructing uncontrollably. A biomedical formulation asks whether a coating can preserve dispersion during storage and circulation but still permit the desired degradation or cargo release. The experimental timescale is different, but the materials question is the same.

This common language suggests useful cross-fertilization. Reversible passivation developed for electronics or catalysis could help manufacture biomedical BP under ambient conditions. Operando spectroscopy developed for battery interfaces could be adapted to follow BP oxidation under catalytic bias. Polymer and polyphenol coatings developed for nanomedicine may provide aqueous processing routes for energy electrodes. Conversely, the rigorous mass-loading and cycling discipline of battery research could improve the quantitative reporting of repeated photothermal or catalytic use.

The field would also benefit from reference materials. A small number of well-characterized BP batches distributed across laboratories could establish how much variation arises from synthesis versus measurement. Round-robin Raman/XPS/AFM analysis would be particularly valuable because reported P-O_x_ fractions and thicknesses depend on preparation and fitting choices. Such standardization is routine in mature materials fields and would make phosphorene performance claims easier to compare.

## 8. Challenges and Future Directions

### 8.1. Define the Material Before Claiming the Property

The term phosphorene is still used for materials with very different layer numbers, lateral dimensions, oxidation levels, and defect populations. At minimum, future studies should report an AFM thickness distribution, lateral-size distribution, Raman spectra, XPS P 2p fitting with an explicit P-O_x_ fraction, and a description of storage time/atmosphere. For application papers, those measurements should be made on the material as used—not only on a freshly prepared aliquot that never entered the device or biological medium.

Batch-to-batch reproducibility deserves its own data. Three independent syntheses with comparable yield, size, and chemical state are more informative for translation than a single carefully selected microscopy image. For direct-deposition methods, mass loading and spatial uniformity should be measured over the full electrode area. For powder routes, recovery yield after washing and solvent exchange should be reported.

### 8.2. Energy Storage: Use Practical Metrics Earlier

Energy-storage papers should continue moving toward electrode-level and cell-level metrics. For alloying anodes, initial Coulombic efficiency, composite-level capacity, areal loading, electrode density, and full-cell N/P balance are critical. For Li-S catalysts, sulfur loading, electrolyte/sulfur ratio, catalyst mass, areal capacity, and pouch-cell data should accompany rate tests. For supercapacitors, areal and volumetric capacitance and complete-device energy/power values are more meaningful than active-material gravimetric capacitance alone.

Operando characterization is also needed to test whether designed interfaces survive. A P-C bond identified in the pristine composite may not be the same after full sodiation. An SEI that appears stable by impedance may still contain electrically isolated phosphorus domains. Combining operando spectroscopy with post-mortem cross-section imaging and quantitative mass balance would make mechanism claims much stronger.

### 8.3. Catalysis: Identify the Real Active State

Catalysis needs a stronger distinction between BP as an active phase, BP as an electronic promoter, and BP as a precursor to a reconstructed catalyst. Under OER in particular, the operating surface may differ substantially from the synthesized material. In situ Raman, X-ray absorption, ambient-pressure XPS, isotope experiments, and dissolution analysis can clarify whether phosphorus remains in the active interface. Turnover metrics should be normalized to a defensible active-site estimate when possible, and not only catalyst mass or geometric area.

For photocatalysis, incident photon flux, apparent quantum yield, spectral distribution, reactor geometry, and catalyst optical density should be reported. Without these parameters, hydrogen-evolution rates from different laboratories are not directly comparable. Long-duration tests should quantify phosphorus loss or oxidation products in the liquid phase rather than relying only on repeated activity cycles.

### 8.4. Biomedicine: Formulation-Specific Toxicology and Realistic Clearance Studies

Biomedical translation requires a shift from generic statements about biodegradability to formulation-specific pharmacology. The final construct should be characterized in serum and under the intended storage conditions. Protein corona, hydrodynamic size, zeta potential, BP oxidation state, drug leakage, and degradation products should be measured over time. Where NIR irradiation is used, laser wavelength, power density, spot size, treatment duration, and resulting tissue temperature should be reported.

Recent reproductive and hepatic studies [63,64] show why organ-specific endpoints matter. A material that clears from blood can still accumulate transiently in an organ or cross a biological barrier. Studies should include both the therapeutic dose and a justified exposure margin, with biodistribution, fecal/urinary clearance, histology, and functional biomarkers. Sex as a biological variable and longer follow-up periods will also be important for systems intended for repeated dosing.

For localized scaffolds and wound dressings, the exposure problem is different but not absent. Degradation products may enter circulation over weeks. Local phosphorus concentration, systemic phosphorus balance, and scaffold residence time should be measured. This would allow regenerative applications to make a stronger case for safety than systemically injected nanosheet formulations.

### 8.5. A Practical Development Roadmap

**Figure 6:**
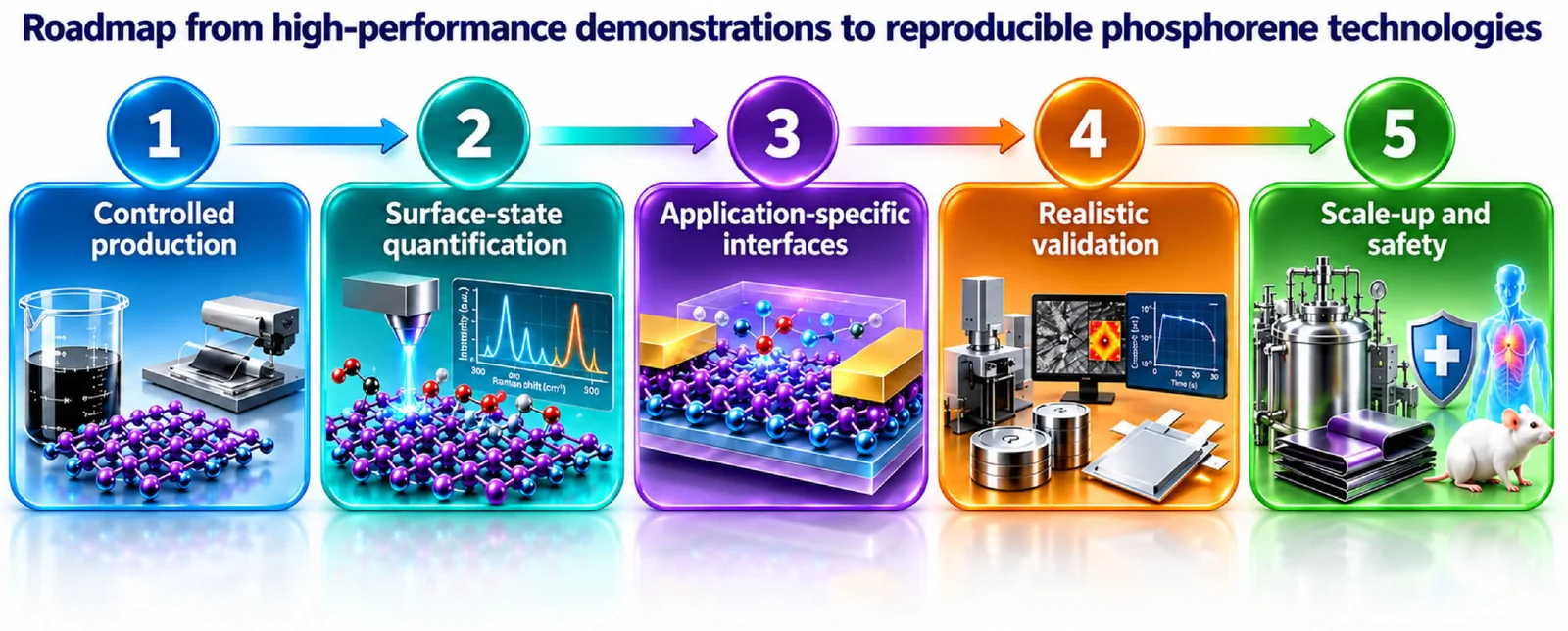
Suggested development sequence for phosphorene technologies. Reproducible production and quantitative surface-state characterization should precede application-specific interface design and realistic validation. Arrows represent a proposed progression of development milestones.

A useful development sequence for phosphorene is shown in Figure 6. First, establish controlled production with a measurable yield and distribution. Second, quantify the surface state before and after storage. Third, create an interface specifically for the target application rather than applying a universal coating. Fourth, validate under realistic operating conditions—high-loading full cells, long-duration catalysis, or clinically relevant dose and exposure. Finally, demonstrate batch reproducibility, scale-up, and safety. Skipping directly from a high-quality TEM image to an application demonstration is increasingly difficult to justify in a mature field.

The strongest opportunity for the next few years may be process integration. Electrochemical exfoliation that simultaneously functionalizes or deposits BP can reduce air exposure and eliminate transfer steps. Removable passivation can separate manufacturing stability from final surface activity. In batteries, direct growth/deposition can lower inactive mass. In catalysis, atomically defined P-M bonding can turn BP from a support into an electronic ligand. In biomedicine, green exfoliation combined with a therapeutic coating can reduce residual-solvent concerns. These are not separate themes; they are manifestations of the same strategy—they control the interface at the moment the nanosheet is created. Table 7 summarizes the cross-application barriers and recommended reporting practices.

## 9. Conclusions

Work published between 2023 and September 2026 shows that black phosphorus and phosphorene research is becoming more disciplined. The field is no longer centered mainly on whether exfoliation is possible; it is increasingly centered on how well the chemical and physical state of the nanosheet can be controlled before, during, and after use. Liquid and electrochemical routes are now routinely paired with functionalization, passivation, direct deposition, or some combination of the three. Molecular overlayers and chelation-based shields can extend stability from days to months, and in some cases they can later be removed to recover surface activity. That is an important shift, because the central challenge of phosphorene has always been the same: it is chemically versatile, but it is also chemically fragile.

In energy storage, the strongest recent studies are not those that simply report another high capacity at low rate. They are the ones that keep electrical contact intact and accommodate phosphorus expansion through covalent bonding, multiscale conductive scaffolds, catalytic interfaces, or binder-free deposition strategies. In catalysis, BP is increasingly used not as an inert support, but as an electronically active interfacial component whose oxidation state and local structure matter. The unresolved issue is to identify the real active phase under HER, OER, photocatalytic H_2_, and CO_2_-reduction conditions. In biomedicine, the field has moved well beyond basic photothermal nanosheets toward targeted delivery systems, regenerative scaffolds, hydrogels, and light-independent antibacterial platforms. At the same time, recent toxicology studies make it clear that biodegradability should not be treated as a substitute for a careful safety assessment.

Going forward, phosphorene research will be judged less by isolated record values and more by reproducibility, context, and durability. A convincing study should define the nanosheet state quantitatively, show how that state changes with time and operation, identify which interface or reconstructed phase is actually responsible for performance, and report boundary conditions that resemble the intended use. For batteries, this means moving earlier toward loading, ICE, areal capacity, electrolyte inventory, electrode density, and full cells; for catalysis, toward photon/active-site normalization and operando chemical-state evidence; and for biomedicine, toward formulation-specific dose, biodistribution, clearance, and organ-level safety. If these requirements accompany continued advances in synthesis and functionalization, phosphorene should remain one of the most versatile 2D material platforms linking energy storage, catalytic conversion, and biomedical engineering.

## Figures and Tables

**Table 1:** Positioning of the present review relative to recent reviews and adjacent 2D-material perspectives.

Review	Primary Scope	Main Strength	Gap Addressed Here
[2] 2023	Battery-anode passivation	BP stability + anode design	Strong on degradation/passivation; limited cross-application comparison
[3] 2026	Synthesis, defects, photocatalysis	Broad materials chemistry	Broad scope; less emphasis on realistic electrochemical/biomedical boundary conditions
[4] 2025	Environmental, energy, biomedical	Cross-application survey	Wide application coverage; interface lifetime is not the organizing axis
[5] 2025	Cancer nanoplatforms	Biomedical/cancer	Deep therapeutic focus; not energy/catalysis
[6] 2024	Drug delivery, imaging, tissue engineering	Biomedical	Application-focused; limited organ-specific toxicology comparison
[7] 2025	Energy-storage devices	Batteries + supercapacitors	Strong device coverage; limited catalytic/biomedical interface comparison
[8] 2025	Stability modification	Passivation/encapsulation	Strong stability focus; application metrics treated secondarily
[9,10] 2026	Violet P/modern 2D heterostructures	Adjacent 2D-material context	Useful interface/fabrication context but not a BP-specific cross-application framework
Present review	Interface evolution from synthesis to use	Energy storage + catalysis + biomedicine	Quantitative practical metrics, active-state verification, formulation-specific safety, and cross-application interface tradeoffs

**Table 2:** Quantitative structure–property descriptors that should accompany phosphorene performance claims.

Design Variable	Representative Quantitative/Structural Descriptor	Expected Consequence	Minimum Evidence	Examples
Layer number/thickness	Bulk bandgap ~0.3 eV; few-layer/monolayer values can approach ~2 eV	Electronic structure, NIR response, surface-to-volume ratio	AFM + Raman; report distributions, not selected flakes	[1]
In-plane anisotropy	Armchair and zigzag directions are inequivalent	Carrier transport, ion diffusion, strain response	Orientation-resolved transport/polarized Raman where relevant	[1]
Vacancies/grain boundaries	Monovacancies, divacancies, adatoms and local amorphous domains observed	Adsorption/active sites vs. oxidation nucleation	Low-dose HRTEM + defect statistics + chemical-state analysis	[11]
Surface oxidation	P-P and P-O_x_ fractions evolve continuously rather than as pristine/degraded binary states	SEI formation, conductivity, P-O-M bonding, degradation	Quantitative XPS P 2p fitting with storage history	[2,8,11]
Covalent interfaces	P-C, P-N and P-O-M bonding reported in recent composites	Mechanical retention + charge redistribution + altered adsorption	XPS/FTIR/Raman plus cycling or operando validation	[12,13,14,15]
Nanosheet lateral size	Often tens of nm to micrometers depending on route/fractionation	Edge density, colloidal stability, diffusion length, biological uptake	TEM/AFM distributions from multiple regions	[16,17]

**Table 3:** Representative phosphorene synthesis and stabilization advances, with the criterion used to judge product quality or stability.

Year/Strategy	Representative System	Key Outcome	Quality/Stability Criterion	What It Solves	Remaining Issue
2023 LPE [16]	NMP sonication + controlled centrifugation	Nanosheets up to ~5.37 µm lateral size	Size/thickness distribution	Preserves larger sheets	NMP removal; total vs. few-layer yield
2023 electrochemical passivation [20]	Exfoliation + AlO_x_ deposition	Improved ambient/thermal stability; twisted BP	Ambient/thermal retention	Formation + protection	Barrier may alter ion/electron access
2023 covalent functionalization [19]	Cathodic exfoliation + CH_3_I	P-C formation; ~9.7% methylation	Chemical functionalization by ^31^P NMR	Protects fresh surface	Uniformity/electronic impact
2024 benign-solvent LPE [17]	Water/Cyrene vs. NMP	Biocompatible processing; stable dispersions	Dispersion stability + chemical state	Improves downstream compatibility	Oxidation control and concentration
2024 noncovalent passivation [23]	Amphiphilic perylene diimides	Ambient stabilization >30 d	Aging in aqueous/ambient media	Aqueous-compatible molecular film	Coating changes uptake/charge transfer
2025 molecular passivation [24]	Self-assembled ferrocene	<10 nm flakes with little degradation ~25 d	AFM/optical aging + calculations	Simple molecular protection	Band-structure modification
2025 removable shield [25]	Tannic acid + Fe(III) chelation	Ambient protection >5 months; deprotection possible	Ambient aging before/after deprotection	Separates storage stability from active state	Residues/deprotection completeness
2026 direct BPE deposition [21]	Reduction-driven deposition on negative feeding electrode	Binder-free few-layer deposit in DI water	Raman/XPS + areal capacitance	Integrates production and fabrication	Scale, loading uniformity, capture efficiency

**Table 4:** Practical electrochemical metrics reported for selected recent phosphorene-based energy-storage studies. NR = not reported in the cited article or not available from the reported configuration.

Device	System	Loading/Inventory	ICE	Cycling/Capacity	Areal/Practical Metric	Full-Cell/Device Evidence	Interpretive Note
LIB	PANI/(BP-CNT) [29]	NR	NR	1150.7 mAh g^−1^ after 100 cycles	NR	Half-cell	Polymer/CNT buffering; practical loading/ICE still needed
LIB	BP/G/Sn [30]	NR	NR	2056 mAh g^−1^ after 50 cycles at 0.15 A g^−1^; 598.6 mAh g^−1^ after 200 cycles at 2 A g^−1^	NR	Half-cell	High capacity; inactive-component penalty and full-cell validation needed
SIB	BP-HC-46 [12]	NR	~70–90% range reported for BP-HC composites	1842.2 mAh g^−1^ after 50 cycles at 0.1 A g^−1^	NR	Half-cell	P-C/P-O-C bonding; loading and electrode density NR
SIB	BP/MXene/CNT [13]	NR	NR	630.7 mAh g^−1^ after 1400 cycles at 2 A g^−1^; 281.1 mAh g^−1^ after 1000 cycles at 10 A g^−1^	NR	NFPP full-cell demonstrated	Strong long-cycle evidence; cell-level energy accounting still needed
Li-S	c-FBP-NC/S [14]	8.74 mg cm^−2^ S	n/a	74% retention over 1000 cycles at 2 C	7.69 mAh cm^−2^; E/S 5.7 µL mg^−1^	Pouch: 4 mg cm^−2^ S, E/S 3.5 µL mg^−1^; 70%/200 cycles	Among the most application-relevant boundary conditions
Li-S	BP-modified polymer electrolyte [33]	0.3 wt% BP in membrane	n/a	1322 mAh g^−1^ initial; 453 mAh g^−1^ after >100 cycles at 0.05 C, 60 °C	NR	Solid-state cell	Room-temperature and higher-loading validation needed
Supercapacitor	BP/c-C_3_N_4_ [15]	NR	n/a	42.1 F cm^−3^; 30,000 cycles	Volumetric metric reported	Flexible device	Appropriate device-level volumetric metric
Supercapacitor	BPE-BP [21]	Directly deposited; mass loading NR	n/a	~11 mF cm^−2^ at 2 mV s^−1^	Areal capacitance	Binder-free symmetric electrode	Capture efficiency/loading uniformity need reporting

**Table 5:** Representative 2023–2026 phosphorene-based catalytic systems and the evidence needed to identify the operating active state.

Reaction	Catalyst/Interface	Representative Metric	Mechanistic Emphasis	Active-State/Normalization Evidence	Critical Issue
HER/OER [37]	1.5 wt% Ni-functionalized BP	136 mV HER/230 mV OER at 10 mA cm^−2^; ~50 h	Ni-P covalent bonding and charge redistribution	Operando/post-test chemistry; loading-normalized activity	Confirm active-state persistence at longer times
HER [38]	N-CoP on ammoniated BP	90 mV at 10 mA cm^−2^	Heterointerface electron transfer and H* binding	Operando/post-test chemistry; loading-normalized activity	Separate CoP and BP contributions
OER [39]	Nickel cobaltite/BP	~197 mV at 10 mA cm^−2^	Interfacial electronic modulation	Operando/post-test chemistry; loading-normalized activity	Operando reconstruction
OER [40]	O-vacancy NiFe_2_O_4_/BP	243 mV at 10 mA cm^−2^; ~100 h	P-Ni/P-O-M bonding; vacancy synergy	Operando/post-test chemistry; loading-normalized activity	Oxidation state during long-term OER
Photocatalytic H_2_ [42]	BP/TpPa-1-COF p-n junction	456.7 µmol h^−1^ g^−1^	2D/2D charge separation	Photon flux/AQY + reactor conditions + post-test chemistry	Quantum yield and long-duration stability
Photocatalytic H_2_ [43]	TiO_2_/BP/CoP	Strong rate enhancement vs. TiO_2_	BP promotion + CoP cocatalyst	Photon flux/AQY + reactor conditions + post-test chemistry	Cross-lab comparison of light intensity
CO_2_RR theory [45]	Monovacancy BP	Predicted enhanced CO-CO coupling	Vacancy-driven potential gradient	Carbon balance/isotope controls + active-site normalization	Experimental defect control
CO_2_RR [47]	NH_2_-UiO-66/BP heterojunction	Enhanced CO formation	Robust interfacial coupling and intermediate stabilization	Carbon balance/isotope controls + active-site normalization	Active-site normalization and BP lifetime

**Table 6:** Formulation-dependent comparison of selected biomedical phosphorene studies. NR = not reported or not sufficiently specified for cross-study comparison.

Application	Formulation/Coating	Size/Thickness	Dose/Route	Biodistribution/Clearance Evidence	Safety/Translation Endpoint
Ischemic stroke [51]	BP@PDA + pterostilbene	NR	Systemic; study-specific dose	BBB transport demonstrated	Barrier opening/off-target exposure; long-term clearance NR
Cancer [50]	BP niosomes + ICG/DOX	NR	Study-specific; formulation dose	Tumor-targeted therapeutic study	Disentangle BP/ICG/DOX contributions; long-term organ clearance NR
Bone [53]	VEGF-BP + DNA hydrogel + 3D scaffold	Scaffold-embedded	Local implantation	Local regenerative model	Long-term degradation and phosphorus mass balance
Diabetic wound [56]	BP hydrogel triboelectric patch	Scaffold-embedded	Local topical/device	Localized exposure	Thermal dose + device durability + systemic degradation products
Diabetic wound [57]	DFO-BP@PDA in GelMA	Scaffold-embedded	Local	Localized exposure	Repeated irradiation + degradation products
Antibacterial [59]	Tannic-acid-armored BP	Nanosheet formulation; exact size study-specific	Local/systemic infection models	Therapeutic efficacy demonstrated	Selectivity across tissues/microbiomes; persistence/clearance
Endothelial toxicity [60]	BP nanosheets	Study-specific	In vitro exposure	n/a	Oxidative-stress/apoptosis threshold; formulation dependence
Reproductive toxicity [63]	Repeated BPNS exposure	Study-specific	Repeated systemic exposure	Blood–testis barrier penetration	Sperm/endocrine/testicular effects; therapeutic-dose relevance
Hepatic metabolism [64]	BP nanomaterials	Study-specific	Repeated exposure	Hepatic exposure	Oxidative stress, mitochondrial dysfunction, lipid disorder

**Table 7:** Cross-cutting barriers and recommended reporting practices.

Recommended Minimum Evidence	Biomedicine	Catalysis	Energy Storage	Barrier
Time-resolved XPS/Raman + storage history	Changed uptake, ROS, degradation	Reconstruction or loss of P sites	SEI growth, impedance, loss of active P	Oxidation/aging
TEM/AFM + statistically meaningful distributions	Faster degradation or ROS	Activity/selectivity changes	Irreversible electrolyte reaction	Defect variability
Operando/in situ spectroscopy where feasible	Coating changes biological identity	Unclear real active state	Unknown bonding after cycling	Interface ambiguity
Areal/full-cell metrics; photon/active-site normalization; dose justification	Non-clinical concentrations	Poorly normalized rates	Low loading half-cell data	Unrealistic metrics
At least three independent batches with yield/composition	Sterility, solvent and formulation consistency	Batch-dependent metal/P ratio	Nonuniform loading; inactive mass	Scale-up
Mass balance and exposure-specific risk assessment	Organ-specific toxicity and clearance	P/metal leaching	Electrolyte and P-containing waste	Safety/environment

## Data Availability

No new data were created or analyzed in this study. Data sharing is not applicable to this review article.

## Abbreviations

BP	Black phosphorus
LPE	Liquid-phase exfoliation
BPE	Bipolar electrochemistry/bipolar exfoliation
LIB	Lithium-ion battery
SIB	Sodium-ion battery
Li-S	Lithium-sulfur
SEI	Solid-electrolyte interphase
HER	Hydrogen evolution reaction
OER	Oxygen evolution reaction
CO_2_RR	Carbon dioxide reduction reaction
PTT	Photothermal therapy
PDT	Photodynamic therapy
ROS	Reactive oxygen species
NIR	Near-infrared
CNT	Carbon nanotube
MOF	Metal–organic framework

## References

[1] Rabiei Baboukani A., Khakpour I., Drozd V., Wang C. (2021). Liquid-Based Exfoliation of Black Phosphorus into Phosphorene and Its Application for Energy Storage Devices. Small Struct..

[2] Mansouri Z., Yaden A., El Manjli F., Ben Ali M., Tiouitchi G., Naamane S., Mounkachi O., El Maalam K. (2023). Peering into Recent Advances in Passivation Strategies of Few-Layer Black Phosphorene toward Battery Anodes: A Review. J. Power Sources.

[3] Hayat A., Emara A.M., Orooji Y., Alghamdi M.M., El-Zahhar A.A., Abu-Dief A.M., Hassan H.M.A., Guo S.-R., Ali H. (2026). Recent Advances in Black Phosphorus: Synthesis, Characterization, Surface Functionalization, Defect Tailoring and Photocatalytic Applications. Renew. Sustain. Energy Rev..

[4] Mishra S.R., Gadore V., Chavda V., Panda S., Roy S., Sahoo P., Pradhan L., Rai H., Pandey S.S., Ahmaruzzaman M. (2025). Black Phosphorene: A Versatile Allotrope Revolutionizing Environmental, Energy, and Biomedical Applications. Coord. Chem. Rev..

[5] Bigham A., Serrano-Ruiz M., Caporali M., Fasolino I., Peruzzini M., Ambrosio L., Raucci M.G. (2025). Black Phosphorus-Based Nanoplatforms for Cancer Therapy: Chemistry, Design, Biological and Therapeutic Behaviors. Chem. Soc. Rev..

[6] Islam M.S., Islam M.T., Hossain M.R. (2024). Phosphorene: A Novel Nanomaterial Revolutionizing Biomedicine. JCIS Open.

[7] Ramachandran T., Kalla R.M.N., Kumar Y.A., Ghosh A., Al-Sehemi A.G., Khan R., Lee J. (2025). Black Phosphorus as a Multifunctional Electrode Material for All Energy Storage Devices. J. Alloys Compd..

[8] Zhang H., Shan C., Wu K., Pang M., Kong Z., Ye J., Li W., Yu L., Wang Z., Pak Y.L. (2025). Modification Strategies and Prospects for Enhancing the Stability of Black Phosphorus. ChemPlusChem.

[9] Nawaz M.Z., Jiang X., Ahmad W., Li M., Zeng Y., Yang X., Li P. (2026). Violet Phosphorus: Synthesis, Fundamental Properties, and Emerging Applications. Appl. Phys. Rev..

[10] Nawaz M.Z., Ahmad W., Hasan I.M.U., Gulzar H., Hasan W.U., Younis U., Farooq A., Saleem M.S., Li M., Chu P.K. (2026). Modern 2D Heterostructures: From Fabrication Challenges to Emerging Applications. Small Methods.

[11] Rabiei Baboukani A., Aghaei S.M., Khakpour I., Drozd V., Aasi A., Wang C. (2022). Defects Investigation of Bipolar Exfoliated Phosphorene Nanosheets. Surf. Sci..

[12] Ai W., Li L., Dong S., Ji H., Liu Y., Zu L., Lian H. (2024). High-Performance Sodium-Ion Anode Based on Stable Phosphorus-Carbon Bond in Black Phosphorus-Hard Carbon Nanocomposite. J. Power Sources.

[13] Ai W., Yang J., Lv J., Zheng Y., Wang R., Liu Y., Zu L., Lian H. (2025). Stabilizing Black Phosphorus-Based Anodes with MXene and Carbon Nanotubes for High-Rate Sodium-Ion Batteries. J. Power Sources.

[14] Zhou J., Shu C., Cui J., Peng C., Liu Y., Hua W., Simonelli L., Wu Y., Dou S.X., Tang W. (2024). Metal-Free Two-Dimensional Phosphorene-Based Electrocatalyst with Covalent P-N Heterointerfacial Reconstruction for Electrolyte-Lean Lithium-Sulfur Batteries. Carbon Energy.

[15] Xin X., Xu Y., Wuliji H., Sun F., Liu Q., Wang Z., Wei T.-R., Zhao X., Song X., Gao L. (2023). Covalently Assembled Black Phosphorus/Conductive C_3_N_4_ Hybrid Material for Flexible Supercapacitors Exhibiting a Superlong 30,000 Cycle Durability. ACS Nano.

[16] Sarwar H., Ji P., Shafique S., Wang X., Yang S. (2023). Production of High-Quality and Large Lateral-Size Black Phosphorus Nanoparticles/Nanosheets by Liquid-Phase Exfoliation. RSC Adv..

[17] Caponio C., Costanzo A., Coiai S., Cicogna F., Pitzalis E., Borsacchi S., Lorenzetti G., Bramanti E., Papalini A., Battisti A. (2024). Cyrene- and Water-Based Exfoliation of Black Phosphorus for Potential Nanolayer-Mediated Disaggregation of Insulin Fibrils. FlatChem.

[18] Rabiei Baboukani A., Khakpour I., Drozd V., Allagui A., Wang C. (2019). Single-Step Exfoliation of Black Phosphorus and Deposition of Phosphorene via Bipolar Electrochemistry for Capacitive Energy Storage Application. J. Mater. Chem. A.

[19] Kuchkaev A.M., Kuchkaev A.M., Sukhov A.V., Saparina S.V., Brylev K.A., Sinyashin O.G., Yakhvarov D.G. (2023). In-Situ Electrochemical Exfoliation and Methylation of Black Phosphorus into Functionalized Phosphorene Nanosheets. Int. J. Mol. Sci..

[20] Wu B., Dekanovsky L., Luxa J., Roy P.K., Hou G., Liao L., Pastika J., Sofer Z. (2023). One-Step Electrochemical Synthesis of AlOx-Passivated Twisted-Phosphorene Nanosheets for Potentially Stable Energy Storage Devices. ACS Appl. Nano Mater..

[21] Rabiei Baboukani A., Qin J., Khakpour I., Allagui A., Pala N., Wang C. (2026). One-Step Bipolar Electrochemical Exfoliation and Deposition of Phosphorene Nanosheets on Negative Feeding Electrode for Binder-Free Capacitive Electrodes. J. Mater. Chem. A.

[22] Rabiei Baboukani A., Khakpour I., Wang C. (2020). Bipolar Exfoliation of Black Phosphorus into Phosphorene. U.S. Patent.

[23] Eisenkolb L., Lloret V., Zink-Lorre N., Pla S., Abellán G., Sastre-Santos Á., Hauke F., Fernández-Lázaro F., Hirsch A. (2024). Investigations of Crucial Factors for the Non-Covalent Functionalization of Black Phosphorus Using Perylene Diimide Derivatives for the Passivation of BP Nanosheets. Chem. Eur. J..

[24] Mu L., Gao S., Jiang J., Wang M., Chen L., Sheng S. (2025). Toward Surface Passivation of Black Phosphorus via a Self-Assembled Ferrocene Molecular Layer. Langmuir.

[25] Guo T., Huang Y., Yu R., Lin S., Huang R., Theato P., Shang J. (2025). Black Phosphorene with Removable Surface Protective Passivation via Covalent Functionalization and Chelation-Based Shield. Small.

[26] Zhao J., Zhao P., Du G., Li S., Fan X., Xie D., Zhu Y., Mei Y. (2025). A Vacancy-Rich Sn-P-I Catalyst for Highly Efficient Black Phosphorus Preparation and Catalytic Mechanistic Investigation. Green Chem..

[27] Rabiei Baboukani A., Khakpour I., Adelowo E., Drozd V., Shang W., Wang C. (2020). High-Performance Red Phosphorus-Sulfurized Polyacrylonitrile Composite by Electrostatic Spray Deposition for Lithium-Ion Batteries. Electrochim. Acta.

[28] Allagui A., Rabiei Baboukani A., Elwakil A.S., Wang C. (2021). Electrochemical Stability Analysis of Red Phosphorus-Based Anode for Lithium-Ion Batteries. Electrochim. Acta.

[29] Yuan S., Sun G., Hong J., Jin H. (2024). Black Phosphorus/PANI/Carbon Nanotube Composite as a Lithium-Ion Battery Anode. ACS Appl. Nano Mater..

[30] Yuan T., Cui X., Li J., Ai W., Gao X., Liu L., Hu F., Cui X., Zu L., Lian H. (2023). A High-Capacity Black Phosphorus-Graphite-Sn Anode for Lithium-Ion Batteries. Mater. Lett..

[31] Chen Y., Ma C., Li Z., Zhang T., Li C., Niu J., Yao S. (2025). Black Phosphorous Nanosheets@CNTs Hybrid Composite for Boosting Conversion of Polysulfides in Lithium Sulfur Batteries. Electrochim. Acta.

[32] Dong X., Chen X., Tong X., Liu G., Yu H., Liu W., Li H., Sun J., Gu X., Zhang S. (2025). Red/Black Phosphorus Heterostructure Anchored on Graphene for Lithium-Sulfur Batteries with Accelerated Conversion Kinetics and Improved Long-Term Stability. Chem. Eng. J..

[33] Li Y., Wang C., Zhu Y., Xie D., Chen X., Mei Y. (2026). Black Phosphorene Modified Polymer Solid Electrolyte Membrane toward Advanced All Solid-State Li-S Batteries. J. Energy Storage.

[34] Halder P., Mondal I., Kundu M., Ghosh A., Paul B.K., Biswas S., Sau S., Chattopadhyay B., Mondal D., Das S. (2024). Optimizing Phosphorene Nanosheets for High Performance All-Solid Asymmetric Supercapacitors: A Theoretical and Experimental Insight. J. Energy Storage.

[35] Zhang X., Wang H., Hong C., He S., Song H., Chu X., Han T. (2023). The Layered Black Phosphorus Modified by Heteroatomic Carbon Microspheres for Energy Storage. Colloids Surf. A.

[36] Radhakrishnan S., Kuniyil N., Sharma A., Rout C.S. (2023). Hierarchal Growth of Integrated n-MoS2/p-Black Phosphorus Heterostructures for All-Solid-State Asymmetric Supercapacitor Electrodes. Energy Fuels.

[37] Zhai W., Chen Y., Liu Y., Ma Y., Vijayakumar P., Qin Y., Qu Y., Dai Z. (2024). Covalently Bonded Ni Sites in Black Phosphorene with Electron Redistribution for Efficient Metal-Lightweighted Water Electrolysis. Nano-Micro Lett..

[38] Fang L., Xie Y., Xu F., Wang M., Wang G. (2023). Nitrogen Doped CoP on Ammoniated Black Phosphorus Nanosheets Enabling Highly Efficient Hydrogen Evolution Electrocatalysis. Dalton Trans..

[39] Jiang W., Tao Y., Ma J., Liu X., Zhu S., Hao W., Xu Q., Fan J. (2023). Interfacial Electronic Modulation on Nickel Cobaltite/Black Phosphorus Heterostructures for Boosting the Electrocatalytic Oxygen Evolution Reaction. ACS Sustain. Chem. Eng..

[40] Mao Y., Wang X., Wu J., Min Y., Li Q., Xu Q. (2024). Enhance the Performance of OER Electrocatalyst via Synergistic Oxygen Vacancies and NiFe_2_O_4_-Phosphorene Heterostructure. J. Alloys Compd..

[41] Kang Y., Wang S., Li J. (2024). Interfacial P-O Covalent Bonds Regulated Electronic Configuration in Black Phosphorus for Improving Oxygen Evolution Activity. Int. J. Hydrogen Energy.

[42] Yan M., Jiang F., Wu Y. (2023). Metal-Free 2D-2D Black Phosphorus/Covalent Organic Framework p-n Heterojunction for Efficient Visible-Light-Driven Hydrogen Evolution without Cocatalysts. Int. J. Hydrogen Energy.

[43] Provinciali G., Filippi J., Lavacchi A., Caporali S., Banchelli M., Serrano-Ruiz M., Peruzzini M., Caporali M. (2023). Black Phosphorus as Promoter for Noble Metal-Free Photocatalytic Hydrogen Production. ChemCatChem.

[44] Wang X., Ma M., Zhao X., Jiang P., Wang Y., Wang J., Zhang J., Zhang F. (2023). Phase Engineering of 2D Violet/Black Phosphorus Heterostructure for Enhanced Photocatalytic Hydrogen Evolution. Small Struct..

[45] Fan J., Wang X., Ma J., Liu X., Wu J., Liu Y. (2024). Efficient Photoreduction of Carbon Dioxide to Acetaldehyde on Monatomic Defective Black Phosphorus. Mol. Catal..

[46] He M., Tian Z., Lin H., Wang G. (2024). Dual-Atom P-Co-Dy Charge-Transfer Bridge on Black Phosphorus for Enhanced Photocatalytic CO_2_ Reduction. Small.

[47] Duan Z., Batur J., Zhang H., Li Y., Li R., Zhang G. (2026). Aerogel-Inspired Interfacial Engineering of Metal-Organic Framework and Black Phosphorus Heterojunction for Enhanced CO_2_ Photocatalytic Reduction. Chem. Commun..

[48] Xiang Y., Gao Y., Yang S., Zhang J., Yang Y. (2024). Folic-Acid-Terminated Black Phosphorus Nanosheets for Targeted Gene Delivery and Photothermal Therapy against Hepatocellular Carcinoma. ACS Appl. Nano Mater..

[49] Qin P., Pang Y., Kong L., Lv Y., Du H., Zhou Y., Wang J. (2024). Cell Membrane Camouflaged Biomimetic Nanocomplex Based on Black Phosphorus Nanosheets to Modulate T Cells for Melanoma Immunotherapy. ACS Appl. Nano Mater..

[50] Kumar R., Singh S.K., Srivastava R.K., Mallick S., Vankayala R. (2025). NIR-Responsive Black Phosphorus Nanosheet-Integrated Niosomes for Combinatorial Chemo-Phototherapy of Cancers. ACS Bio Med Chem Au.

[51] Yin S., Hou J., Li J., Zeng C., Chen S., Zhang H., Tian X. (2024). Polydopamine-Modified Black Phosphorus Nanosheet Drug Delivery System for the Treatment of Ischemic Stroke. Regen. Biomater..

[52] He Y., Yang X., Bai Y., Ba X., Ye T., Zhong Z., Chen Z., Li H., Tang K. (2026). Bionic Black Phosphorus Nanosheets Confer Dual Protection against Nephrocalcinosis-Induced Kidney Injury via ROS Scavenging and NETosis Inhibition. Mater. Today Bio.

[53] Miao Y., Chen Y., Luo J., Liu X., Yang Q., Shi X., Wang Y. (2023). Black Phosphorus Nanosheets-Enabled DNA Hydrogel Integrating 3D-Printed Scaffold for Promoting Vascularized Bone Regeneration. Bioact. Mater..

[54] Li H., Xue Y., Zhang T., Huang Y., Xu Y., Dai Y., Dong Y., Lyu F., Li L. (2025). Black Phosphorus Nanosheets Incorporated Mesenchymal Stem Cell Spheroids for Accelerated Bone Regeneration. ACS Appl. Nano Mater..

[55] Wang J., Han Y., Liu M., Yang C., Xu X., Lin C., He C., Zhu Y., Wang J., Huang X. (2025). Zinc-Doped Black Phosphorus Nanosheets Integrated into Janus Stratified Structural Hydrogel for Vascularized Bone Regeneration. ACS Biomater. Sci. Eng..

[56] Ge X., Mao M., Yu H., Liu J., Kong J., Sun Y., Huang W., Wang D.Y., Wang Y. (2024). Application of Self Powered Black Phosphorus Nanosheets-Based Hydrogel Skin Patch in Wound Healing of Diabetes. Nano Energy.

[57] Li Y., Pan Y., Kong L., Long H., Teng J., Zhen H., Ding Q., Pan R., Tian X. (2025). Black Phosphorus Nanosheet Hydrogels Elicit a Thermogenic Effect and Enhance Diabetic Wound Healing through Controlled Drug Release. Mater. Des..

[58] Shen J., Jia G., Wu Q., Yang H., Jiang Y., Wu X., Chai Y., Zhang C., Xu J. (2025). Black Phosphorus Nanosheets Fortified with Catalase to Enhance Schwann Cell Responses for Neural Repair. J. Control. Release.

[59] Xu Y., Zhang J., Li L., Zhang Y., Chen S., Du W., Luo W., Meng X., Hu X., Wang Y. (2026). Curvature-Selective Antibacterial Potency of Tannic Acid-Armored Black Phosphorus Nanosheets against Multispecies Biofilms and Resistant Systemic Infections. ACS Nano.

[60] Dong H., Wen Y., Lin J., Zhuang X., Xian R., Li P., Li S. (2023). Cytotoxicity Induced by Black Phosphorus Nanosheets in Vascular Endothelial Cells via Oxidative Stress and Apoptosis Activation. J. Funct. Biomater..

[61] Fan Z., Wu S., An Z., Wang Y., Xu B., Wang X., Xu Y., Li H., Duan G., Zhang S. (2024). Unraveling the Stress-Induced Toxicity of Black Phosphorus Nanosheets and the Underlying Mechanism. Colloid Interface Sci. Commun..

[62] Zhang B., Xing Y., Guo Y., Jin S., Shao X., Fan J., Zhang C., Wang Y. (2024). Protective Role of Melatonin against Liver Toxicity from Black Phosphorus Nanosheets via Oxidative Stress and Pyroptosis. ACS Omega.

[63] Jiang S., Ruan F., Zhang F., Ding X., Long Z., Zhu S., Fu Z., Zuo Z., Jiang X., He C. (2026). Black Phosphorus Nanosheets Penetrate the Blood-Testis Barrier and Induce Reproductive Toxicity in Male Mice. Environ. Health.

[64] Zhu S., Ruan F., Zhang Y., Leng Y., Wu Y., Sun Y., Yang C., Zuo Z., Chen S. (2026). Black Phosphorus Nanomaterials Induce Hepatic Lipid Metabolism Disorders by Triggering Oxidative Stress and Mitochondrial Dysfunction. Part. Fibre Toxicol..

